# Microenvironment characterization and multi-omics signatures related to prognosis and immunotherapy response of hepatocellular carcinoma

**DOI:** 10.1186/s40164-020-00165-3

**Published:** 2020-05-25

**Authors:** Furong Liu, Lu Qin, Zhibin Liao, Jia Song, Chaoyi Yuan, Yachong Liu, Yu Wang, Heze Xu, Qiaofeng Zhang, Youliang Pei, Hongwei Zhang, Yonglong Pan, Xiaoping Chen, Zhanguo Zhang, Wanguang Zhang, Bixiang Zhang

**Affiliations:** 1 Hepatic Surgery Center, Tongji Hospital, Tongji Medical College, Huazhong University of Science and Technology, 1095 Jiefang Avenue, Wuhan, 430030 Hubei China; 2 Hubei Province for the Clinical Medicine Research Center of Hepatic Surgery, Wuhan, 430030 Hubei China; 3 Department of Anesthesiology, Union Hospital, Tongji Medical College, Huazhong University of Science and Technology, Wuhan, 430022 China; 4 Department of Gynecology and Obstetrics, Tongji Hospital, Tongji Medical College, Huazhong University of Science and Technology, Wuhan, 430030 China; 5 The Second Clinical Medicine College, Tongji Medical College, Huazhong University of Science & Technology, Wuhan, China

**Keywords:** Hepatocellular carcinoma, Immune subtypes, Multi-omics signatures, Immunotherapy, MMP9

## Abstract

**Background:**

Immune cell infiltration in the tumor microenvironment (TME) affects tumor initiation, patients’ prognosis and immunotherapy strategies. However, their roles and interactions with genomics and molecular processes in hepatocellular carcinoma (HCC) still have not been systematically evaluated.

**Methods:**

We performed unsupervised clustering of total 1000 HCC samples including discovery and validation group from available public datasets. Immune heterogeneity of each subtype was explored by multi-dimension analysis. And a support vector machine (SVM) model based on multi-omics signatures was trained and tested. Finally, we performed immunohistochemistry to verify the immune role of signatures.

**Results:**

We defined three immune subtypes in HCC, with diverse clinical, molecular, and genomic characteristics. Cluster1 had worse prognosis, better anti-tumor characteristics and highest immune scores, but also accompanied by immunosuppression and T cell dysfunction. Meanwhile, a better anti-PD1/CTLA4 immunotherapeutic response was predicted in cluster1. Cluster2 was enriched in TAM-M2 and stromal cells, indicating immunosuppression. Cluster3, with better prognosis, had lowest CD8 T cell but highest immune resting cells. Further, based on genomic signatures, we developed an SVM classifier to identify the patient’s immunological status, which was divided into Type A and Type B, in which Type A had poorer prognosis, higher T cell dysfunction despite higher T cell infiltration, and had better immunotherapeutic response. At the same time, MMP9 may be a potential predictor of the immune characteristics and immunotherapeutic response in HCC.

**Conclusions:**

Our work demonstrated 3 immune clusters with different features. More importantly, multi-omics signatures, such as MMP9 was identified based on three clusters to help us recognize patients with different prognosis and responses to immunotherapy in HCC. This study could further reveal the immune status of HCC and provide potential predictors for immune checkpoint treatment response.

## Introduction

Hepatocellular carcinoma (HCC), the main histology type (70–90%) of liver cancer, ranks sixth in cancer incidence and fourth in death. In recent years, the incidence of HCC has increased in most regions of the world and decreased in some countries in Asia [1, 2]. Currently, the main treatment for HCC patients in early stages is surgery, combination with transarterial chemoembolization, ablation and liver transplantation [3]. For others in advanced stages, the effective approaches involve molecular targeting agents (tyrosine kinase inhibitors: sorafenib, lenvatinib and regorafenib) [4], and huaier granule, a traditional Chinese medicine [5]. Although these methods have improved the prognosis of HCC patients, the overall survival of HCC remains challenging for the heterogeneity of HCC [1, 6].

Immunotherapy has been a growing focus because of its effectiveness in many tumors [7, 8], Particularly, targeted therapies for immune checkpoints such as anti-CTLA4 and anti-PD1 have benefited a part of patients with solid tumors [9–12], although not all patients show the response to immunotherapy [13]. Even though clinical trials are under way, the future of immunotherapy in HCC is uncertain. In chronic hepatitis caused by viral infection (HBV, HCV), alcoholism, metabolic diseases (non-fatty liver disease), and drug damage (aflatoxin, aristolochic acid), changes in the liver microenvironment and imbalance in the proportion of immune cells eventually lead to immune escape and the promotion of HCC [14, 15]. The role of different components (including tumor-associated macrophages (TAM), myeloid-derived suppressor cells (MDSC), regulatory T cells (Tregs), CD8+ cytotoxic T lymphocytes, fibroblasts) of tumor microenvironment (TME) in hepatocellular carcinoma has also been discussed in many studies [16]. Determining TME of patients with cancers before treatment can demonstrate the immune status to predict the prognosis of patients and the response to chemotherapy and immunotherapy drugs [17, 18].

To understand the immune microenvironment of HCC, some researches have done to investigate the immune subtypes of HCC [19]. However, there are lack of multi-omics (mRNA, miRNA, long non-coding RNA (LncRNA), somatic mutation, DNA methylation, copy number variations and reverse phase protein array (RPPA)) studying focusing on immune microenvironment characteristics and immunotherapy strategies of HCC.

In this study, we used two computational algorithms to estimate the abundance of 26 TME cells of 1000 HCC samples and performed three clustering methods to confirm 3 clusters, the optimal number of clusters. Then we recognized the differences of immunes cell abundance, immune gene expression, genomic characteristics, molecular and biological function, and clinical outcomes among the three subtypes of HCC. Finally, we developed a support vector machine (SVM) classifier using multi-omics signatures to identify patients with significant prognostic differences and different responses to immunotherapy in HCC, and preliminarily demonstrated that the expression of MMP9 could predict the immune characteristics of HCC.

## Materials and methods

### Patients collection

We systematically collected nine HCC datasets with the number of patients in each set greater than 50 in the three datasets: NCBI GEO, the Cancer Genome Atlas-Liver hepatocellular carcinoma (TCGA-LIHC), the International Cancer Genome Consortium (ICGC). We used four datasets to compare the differences of immune cell abundance between carcinoma and adjacent tissues. TCGA-LIHC dataset (N = 374) was used for discovery cohort to identify the immune subtype of HCC. A meta-validation cohort (N = 626) containing five independent RNA-seq and microarray datasets was used to validate the results. For the details of data, please see Additional file 1: Materials and methods. Another validation cohort of 134 patients, who were diagnosed as HCC by 2 independent pathologists and underwent primary liver cancer resection in Wuhan Tongji hospital from 2014 to 2015, with survival data by telephone follow-up, were included in this study. This research on patients’ tissues was authorized by the by the Ethic Committee of Wuhan Tongji Hospital and received written informed consent from patients.

### Identify immune subtypes in discovery and validation groups

We calculated the relative TME cell abundance of by MCPcounter [20] and CIBERSORT [21]. Three packages (*mclust* [22], *NbClust* [23], and *ConsensusClusterPlus* [24]) were performed to determine the optimal number of clusters both in LIHC and validation cohorts. For the details of processing data, please see Additional file 1: Materials and methods.

### Statistics

Wilcoxon rank-sum test was used to compare two groups of continuously distributed variables. Kruskal–Wallis test was used to compare three or more groups of continuously distributed variables, and Steel–Dwass test was utilized for multiple comparisons of post hoc tests. The survival in different groups was evaluated by Log-Rank test. The categorical variables in contingency tables were compared by Chi-squared test or Fisher’s exact test. The FDR correction was performed in multiple tests.

The correlation coefficients of two variables were calculated by Pearson or Spearman analysis, and |R| ≥ 0.15 was considered to be correlated. All analyses were performed in R software (version: 3.6.1). ns: no significance, *P < 0.05, **P < 0.01, ***P < 0.001.

### Other methods

For the details of other methods and materials, please see the Additional file 1: Materials and methods.

## Results

### The subtypes of immune microenvironment in HCC

**Fig. 1:**
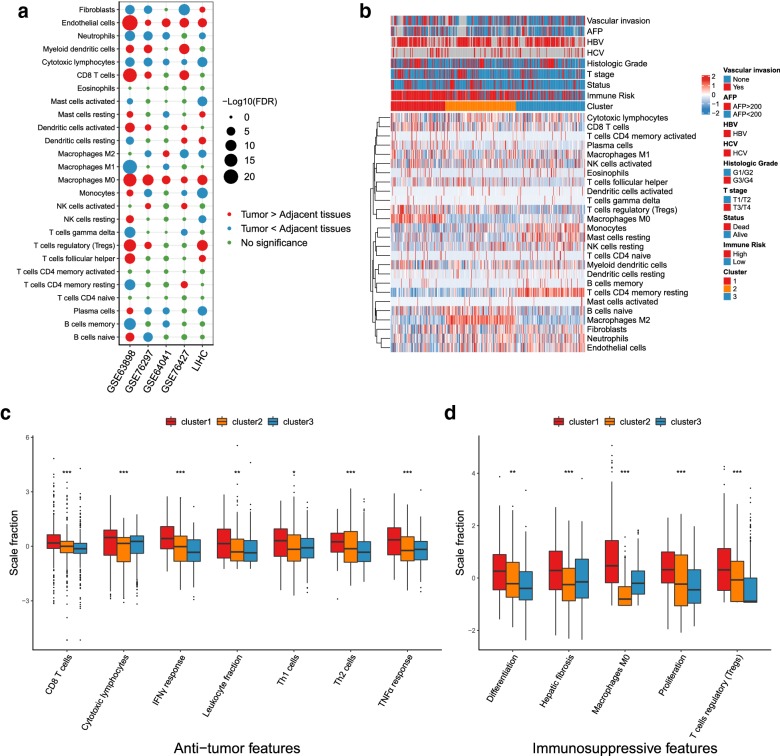
The subtypes of immune microenvironment in HCC. **a** Comparison of TME cells between HCC samples and adjacent tissues in multiple cohorts. Red: The abundance of TME cell is high in HCC tissues; Blue: The abundance of TME cell is low in HCC tissues; Green: No significance between HCC and non-tumor tissues. The size of the bubble means − log_10_ (FDR). Wilcoxon signed rank test was used to compare the significances of TME cell fractions between HCC samples and adjacent tissues. **b** Unsupervised clustering of TME cells in TCGA-LIHC with 374 patients. The representative anti-tumor (**c**) and immunosuppressive (**d**) characteristics among the three clusters. ns: no significance, *P < 0.05, **P < 0.01, ***P < 0.001

The schematic diagram of the whole analysis process is shown in Additional file 3: Fig. S1. Firstly, to find biomarkers and understand the dynamic evolution of immune microenvironment in tumorigenesis, we evaluated the composition of TME cells of both HCC tissues and adjacent tissues in four datasets. The abundance of endothelial cells, myeloid dendritic cells, CD8 T cells, macrophages M0, Tregs and activated dendritic cells were almost consistently higher in tumor tissues, while neutrophils and cytotoxic lymphocytes were lower than adjacent tissues (Fig. 1a). Since the adjacent tissues are hardly normal hepatocyte tissues, but rather comprise chronic hepatitis or cirrhosis tissues, the above-mentioned changes in immune cell composition might play an important role in the transformation of inflammatory status to cancer, such as angiogenesis in tumor [25], immunosuppression of myeloid dendritic cells and macrophages [26, 27].

Then we focused on the immune microenvironment of HCC. After expectation–maximization algorithm and unsupervised K-means clustering were applied to TCGA immune dataset, both methods supported that 3 immune subtypes were identified in 374 HCC samples (Additional file 3: Fig. S2). Similarly, the validation meta-cohort dataset with 626 HCC patients was also determined 3 immune clusters (Additional file 3: Fig. S3). The cluster of each HCC patient in the discovery and validation cohorts could be seen in Additional file 2: Table S2.

Also, we found that under K-means clustering, the same K number in the TCGA and meta-cohort group showed the similar error value change, which revealed the consistency of the two cohorts (Additional file 3: Figs. S2c, S3c). To validate the concordance of the two datasets, we assessed reproducibility of discovery cohort’s subtypes in the independent validation group. The TME cells in the same cluster of the both groups show highly linear correlation (cluster1 is 0.786 (P = 1.99 × 10^−6^), cluster2 is 0.836 (P = 1.03 × 10^−7^), cluster3 is 0.886 (P = 1.706 × 10^−9^), Pearson correlation) (Additional file 3: Fig. S4a).

### Immune microenvironment and biological processes among three HCC subtypes

These three immune subtypes were associated with distinct patterns of immune environment cell abundance. Cluster1 was rich in high infiltration activated adaptive immune cells (CD8 T cells, cytotoxic lymphocytes, T follicular helper cells), tumor-associated macrophage (TAM)-M0, plasma cells, and Tregs, while some innate and inactivated immune cells (resting mast cells, resting memory CD4 T cells, monocytes and neutrophils) and stromal cells (endothelial cells and fibroblasts) tended to decrease. Cluster2 was chartered by high infiltration of stromal cells (endothelial cells and fibroblasts) and TAM-M2, low abundance of cytotoxic lymphocytes and TAM-M0, implying immunosuppressive status. And the abundance of other immune cells in cluster 2 is moderate infiltration between cluster1 and cluster3. Cluster3 showed increased infiltration characteristics on inactivated immune cells (resting memory CD4 T cells, resting mast cells, resting NK cells and resting dendritic cells), stromal cells (endothelial cells and fibroblasts) and monocytes, which were depleted in cluster1. And CD8 T cells, T follicular helper cells, and Tregs were reduced in cluster3 (Fig. 1b, Additional file 3: Fig. S4b).

Considering the heterogeneity of HCC, other immune biological processes would have different impacts on the TME subtypes. We calculated the abundance level of T effector, Th1, Th2, Th17 cells, and found they were highly infiltrated in cluster1 except the Th17 cells (Fig. 1c, Additional file 3: Fig. S5a), which was consistent with the above results and previous reports that Th17 inhibited the anti-tumor effects of Th1 and Th2 cells [16, 28]. On the other hand, we tested immune related gene modules. Cluster1 had a beneficial anti-immune response (higher IFN-γ, TNF-α and DNA damage repair response, leukocyte fraction and lower reactive stroma and angiogenesis), despite high levels of gene modules in hepatic fibrosis, proliferation and differentiation (Fig. 1c, d, Additional file 3: Fig. S5a). And cluster3 exhibited opposite levels. Further, there were no significant difference in the DNA reads of HBV and HCV among the three clusters (Additional file 3: Fig. S5b, c).

These results implied that cluster1 might be in a state with the highest anti-tumor characteristics (leukocyte fraction, cytotoxic CD8 T, Th1, Th2 cells and IFN-γ response) but high immunosuppressive features (Tregs, TAM-M0 and Th17 cells, proliferation and differentiation), cluster2 tended to be an immunosuppressive status, and cluster3 might be an immune resting state.

Given the immunotherapeutic role of CD8 T cells in tumors, cluster1 was classified as “CD8 T cell-hot” and immune-counterbalanced type tumor (with coexisting immune-activation and immune-suppression), cluster3 intended to be “CD8 T cell-cold”, and cluster2 was between other two clusters and immunosuppressive.

### Clinical characteristics of TME cells among subtypes in HCC

**Fig. 2:**
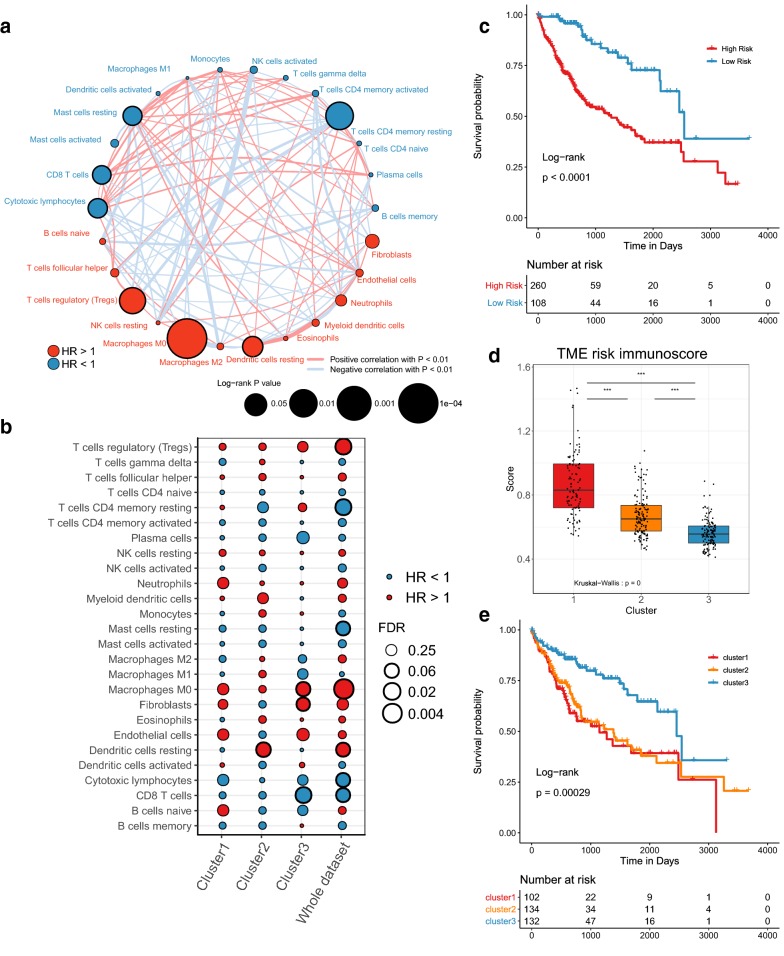
Prognostic evaluation of TME cells in HCC. **a** Network graph of TME cells’ interaction in HCC. The bubble color represents different prognostic effects: Red: high-risk TME cells, Blue: protective immune cells; The size of bubble indicates P-value, and bold edges means significant survival of TME cells (univariate COX regression, P < 0.05); The lines of network represent the correlations among TME cells (Spearman correlation). The thicker the line, the stronger the correlation. The red represents a positive correlation, and the light grey represents a negative correlation. **b** Prognostic analysis of each TME cell for each subtype. **c** Kaplan–Meier overall survival curves based on TME cell LASSO model. High risk and low risk group was divided by the optimal cutoff value by *survminer* package. **d** Distribution of TME immune risk scores based on TME cell LASSO model and **e** Kaplan–Meier OS curves among three subtypes. ns: no significance, *P < 0.05, **P < 0.01, ***P < 0.001

In consideration of the prognosis of TME in tumor, each immune cell was evaluated the effect on prognosis. Four immune cell infiltration (resting memory CD4 T cells, cytotoxic lymphocytes, CD8 T cells, resting mast cells) indicated better overall survival, while Tregs, TAM-M0 and resting dendritic cells were significantly correlated with the poor survival (Log rank test, P < 0.05, Fig. 2a). And we also analyzed the prognosis effect of each immune cell abundance among the 3 immune subtypes. As a whole, each immune cell had similar prognosis effect among 3 subtypes, though their significances varied (Fig. 2b). An immune risk score system, a lasso score model based on TME cells, was established to estimate the immune risk of each patient. We found that patients with high immune risk scores would have worse prognosis, and cluster1 and cluster2 had higher immune risk scores (Fig. 2c, d). Then we focused on the prognosis among the three subtypes. Although cluster1 had highest CD8 T cells infiltration, this cluster had worse overall survival, while cluster3 with lowest CD8 T cells infiltration had better prognosis (Fig. 2e), which seemed paradoxical with previous studies that that “CD8 T cell-hot” type tumor was beneficial to survival [29–32].

Next, we tested the relation between clinicopathologic features and the 3 immune subtypes. Patients in cluster1 had higher α-fetoprotein (AFP), and there existed more patients in advanced stages (T2/T3, Stage II/Stage III) and less in early stage (T1, Stage I) in cluster1 (Table 1; Chi-squared test or Fisher’s exact test, P < 0.05). In addition, we analyzed the influence of three clinical characteristics (AFP, T stage and pathological stage) on the prognosis. T stage and pathological stage showed strong effects on the prognosis (Additional file 3: Fig. S6). The higher the stage, the worse the survival. This result partially explained why cluster1 had worse prognosis.

**Table 1:** Associations between clinical characteristics of HCC patients from TCGA and three immune subtypes

	Cluster1 (n = 104)	Cluster2 (n = 135)	Cluster3 (n = 132)	P-value
Gender				
Female	33 (31.7%)	42 (31.1%)	46 (34.8%)	0.788
Male	71 (68.3%)	93 (68.9%)	86 (65.2%)	
Race				
Asian	52 (50.0%)	52 (38.5%)	54 (40.9%)	0.116
Black	7 (6.7%)	7 (5.2%)	3 (2.3%)	
White	42 (40.4%)	74 (54.8%)	68 (51.5%)	
Alcohol consumption	35 (33.7%)	48 (35.6%)	47 (35.6%)	0.299
HBV	63 (60.6%)	84 (62.2%)	82 (62.1%)	0.172
HCV	21 (20.2%)	22 (16.3%)	18 (13.6%)	0.808
Drug treatment	13 (12.5%)	21 (15.3%)	30 (22.5%)	0.109
AFP				
AFP < 200	47 (45.2%)	70 (51.9%)	84 (63.6%)	*0.018*
AFP > 200	31 (29.8%)	19 (14.1%)	27 (20.5%)	
Vascular invasion				
None	46 (44.2%)	74 (54.8%)	86 (65.2%)	0.057
Yes	38 (36.5%)	33 (24.4%)	38 (28.8%)	
Tumor grade				
G1	9 (8.7%)	30 (22.2%)	16 (12.1%)	0.053
G2	50 (48.1%)	57 (42.2%)	70 (53.0%)	
G3	40 (38.5%)	43 (31.9%)	39 (29.5%)	
G4	5 (4.8%)	3 (2.2%)	4 (3.0%)	
T stage				
T1	38 (36.5%)	62 (45.9%)	81 (61.4%)	*0.010*
T2	34 (32.7%)	36 (26.7%)	24 (18.2%)	
T3	28 (26.9%)	31 (23.0%)	21 (15.9%)	
T4	4 (3.8%)	5 (3.7%)	4 (3.0%)	
M stage				
M0	75 (72.1%)	92 (68.1%)	99 (75.0%)	0.215
M1	1 (1.0%)	0 (0%)	3 (2.3%)	
MX	28 (26.9%)	43 (31.9%)	30 (22.7%)	
N stage				
N0	69 (66.3%)	84 (62.2%)	99 (75.0%)	0.103
N1	2 (1.9%)	2 (1.5%)	0 (0%)	
NX	32 (30.8%)	49 (36.3%)	33 (25.0%)	
Pathological stage				
Stage I	36 (34.6%)	57 (42.2%)	78 (59.1%)	*0.002*
Stage II	32 (30.8%)	32 (23.7%)	22 (16.7%)	
Stage III	32 (30.8%)	31 (23.0%)	22 (16.7%)	
Stage IV	1 (1.0%)	1 (0.7%)	3 (2.3%)	

Italics font of P-value represented P < 0.05

### The immunogenicity and genomic alterations among immune types of HCC

**Fig. 3:**
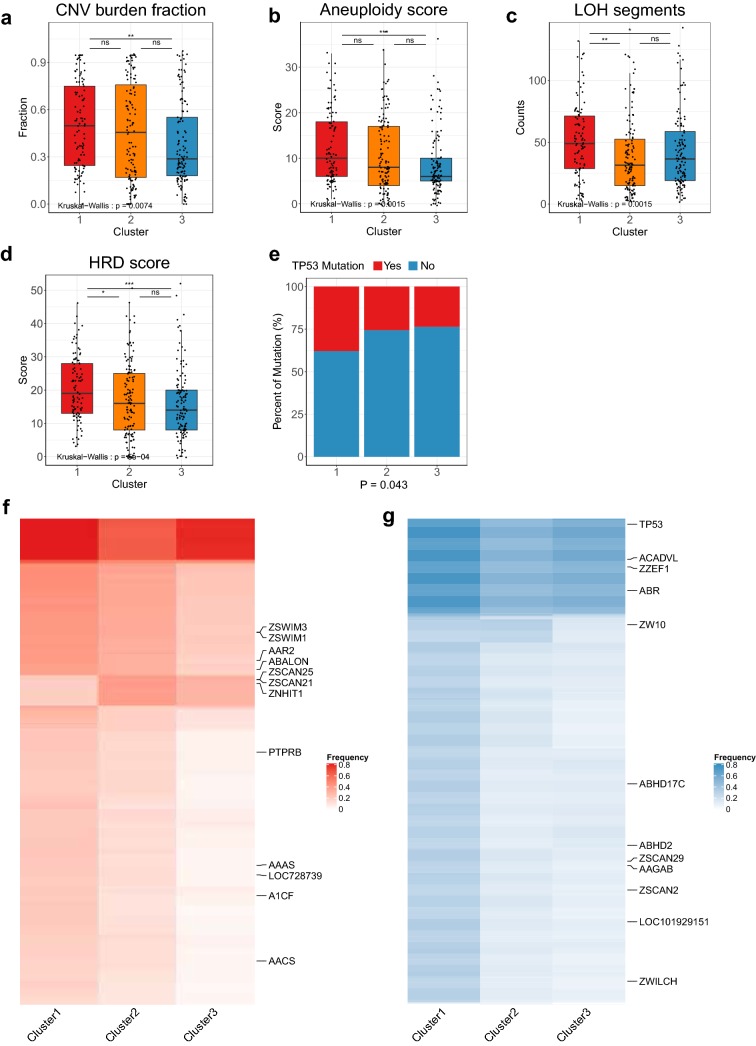
Somatic and immunogenic mutation alterations among three subtypes. Comparison of CNV burden fraction (**a**), Aneuploidy score (**b**), LOH segments (**c**), HRD score (**d**) among three clusters. **e** TP53 mutation frequency among three clusters (Chi-squared test, P < 0.05). Heatmap of significant amplification genes (**f**) and deletion genes (**g**) in each subtype (Chi-squared test or Fisher’s exact test, FDR < 0.1). ns: no significance, *P < 0.05, **P < 0.01, ***P < 0.001

To demonstrate the genomic alterations among three clusters and why cluster1 with the highest abundance of CD8 T cells had poor survival, we analyzed the genomic changes of the three clusters. We could not find significant changes in overall tumor mutation burden (TMB), neoantigen load, indel, immunogenic mutation and immunogenic indel (Additional file 3: Fig. S7a–e), which were potential biomarkers for immunotherapy responsiveness [33–35], indicting other genomic mechanisms affecting all three subtypes. Then, we compared some indicators based on copy number variations (CNVs) and somatic mutations, such as CNV burden, intratumoral heterogeneity (ITH), homologous recombination deficiency (HRD), loss of heterozygosity (LOH) and aneuploidy. In general, cluster1 had highest CNV burden, HRD scores, aneuploidy scores and LOH segments (P < 0.05, Fig. 3a–d, Additional file 3: Fig. S7f). In addition, ITH scores in cluster1 seemed higher, but there was no distinct difference in ITH scores among three clusters (P = 0.112, Additional file 3: Fig. S7g). These data suggested high CNV alterations in cluster1 might cause silenced immune surveillance [36].

Among the genes with significant mutations in HCC (top 15), we found that only TP53 was significant in three clusters (Fig. 3e, Additional file 3: Fig. S7h). Notably, the role of TP53 in TME had been widely researched [37, 38], which further explained the poor prognosis of cluster1. Further, we focused on CNVs of patients with different immune status (Additional file 3: Fig. S7i). Concretely, amplifications in 83 regions including 2033 genes and deletions in 45 regions including 1194 genes were significant among the three cluster (FDR < 0.1, Additional file 2: Tables S3, S4). And most significant regions or genes related amplification or deletion were enriched in cluster1 immune subtype, such as TP53 deletion (Fig. 3f, g), which implied immune escape in cluster1 might be driven by genomic alterations. Overall, these somatic mutations and CNVs in HCC provided new research ideas for the formation of TME in HCC.

### Regulation of immunomodulators and prediction to immune checkpoint blockade therapy

Immunomodulators are crucial in the formation of TME, immune surveillance, immune escape, and immunotherapy [39, 40], Therefore, we detected 76 immunomodulators (14 antigen presentation molecules, 25 inhibitors and 37 stimulators; Additional file 2: Table S5) gene expression under CNVs, epigenetics and miRNA controls.

**Fig. 4:**
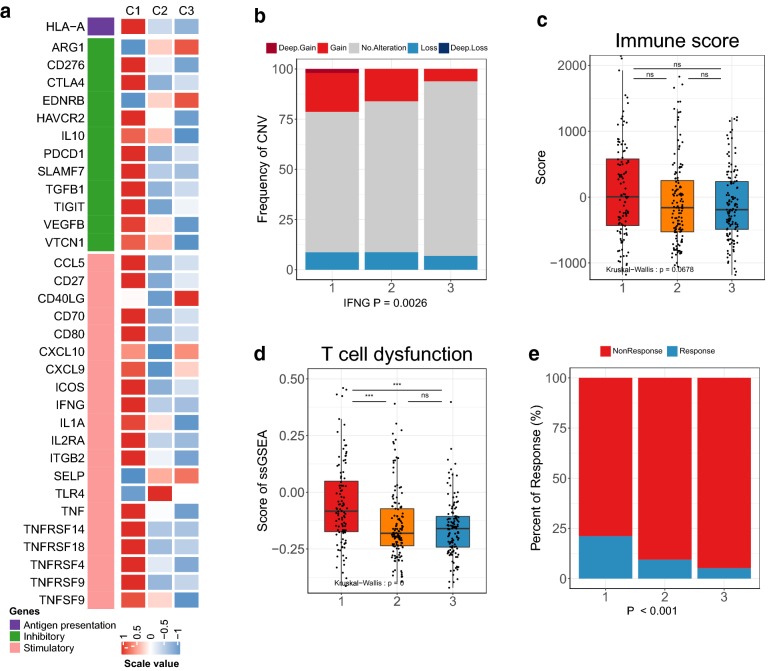
Regulation of immunomodulators and prediction of the response to the immune checkpoint blockade therapy. **a** Heatmap for the significant differential expression of immunomodulators. **b** CNV distribution of IFNG among three clusters. Each column represents the total proportion of each subtype and each color indicates different type of CNV (Chi-squared test). **c** Immune scores, and **d** scores of T cell dysfunction among three subtypes in TCGA-LIHC. **e** Prediction of immunotherapy responsiveness among three clusters in TCGA-LIHC by TIDE (Chi-squared test). ns: no significance, *P < 0.05, **P < 0.01, ***P < 0.001

33 immunomodulators gene expression varied among three subtypes (Additional file 3: Figs. S8–S10). Most genes were highly expressed in cluster1 (Fig. 4a). Promoter methylation of these immunomodulators were negatively correlated with their corresponding mRNA expression, such as TGFB1, PD1, IL10, CTLA4 (Additional file 3: Fig. S11a), which implied the epigenetic cis-regulation of DNA methylation. In another way, we also investigated the way in which miRNA regulated immunomodulators. Negative correlations between expression of miRNAs and immunomodulators revealed the complexity of regulating immunomodulators (Additional file 2: Table S6, Additional file 3: Fig. S11b), which suggested the important roles of miRNA in TME formation.

CNVs of immunomodulators were detected, the amplification frequency of 4 immunomodulators (CD27, IFNG, IL10, IL2RA) and the deletion frequency of 4 immunomodulators (CD276, CXCL10, CXCL9, HLA-A) were significantly different among the three subtypes (Chi-squared test or Fisher’s exact test, P < 0.05). And we noticed that these 8 genes were amplified or deleted most in cluster1 and least in cluster3 (Fig. 4b, Additional file 3: Fig. S11c), indicting the TME modulation differences of the three immune subtypes.

Further, the higher level of most immune-related stimulators implied the activated immune state of cluster1. Then, we evaluated the immune state among three clusters with immune score and stromal score, calculated by *ESTIMATE* algorithm [41] to estimate infiltrating overall immune cells and stromal cells in tumor tissues. Although P value of immune and stromal score in three subtypes was not significant in TCGA-LIHC (P = 0.068 and P = 0.083), cluster1 tended to have higher immune scores and lower stromal scores (Fig. 4c, Additional file 3: Fig. S12a), which was further verified in the meta-validation cohort with significant differences (P < 0.05, Additional file 3: Fig. S12b, c), possibly due to the larger number of patients in the validation cohort (N = 626). These results showed activated immune state of cluster1. However, we also observed that most immune inhibitors (immune checkpoints) were highest in cluster1, which demonstrated that cluster1 might associate with T cell dysfunction and immunosuppression, despite high CD8+ cytotoxic T lymphocytes infiltration. To verify this observation, we evaluated the T cell dysfunction in the TCGA group and validation cohort by scoring the defined gene set (TGFB1, CD274, CTLA4, IL10, PDCD1, TNFRSF9, CD276, HAVCR2, LAG3, TIGIT, ICOS), which had been proven to exhaust T cells [42, 43]. As we referred, cluster1 had the highest scores in both the test and validation cohorts (Fig. 4d, Additional file 3: Fig. S12d, P < 0.001). These results indicated that patients in cluster1 had more T cell exhaustion, and the T cell function of these patients needs to be reinvigorated by immune checkpoint therapy [44].

To further validate this result, we applied the Tumor Immune Dysfunction and Exclusion (TIDE) [45] tool to predict the response of patients to immune checkpoint blockade (CTLA4 and PD1 therapy). We found the overall response in HCC was dissatisfactory, with 42 responders in 374 TCGA patients and 11 responders in 221 validated patients. We evaluated treatment response in each cluster, and most response events occurred in cluster1 (TCGA-LIHC response rate: 22/104 (21.1%) vs. 13/137 (9.5%) vs. 7/133 (5.3%), Chi-squared test, P < 0.001; LICA-FR and GSE64041 cohorts: 11/72 (15.3%) vs. 0/65 (0%) vs. 0/84 (0%), Fisher’s exact test, P < 0.001; Fig. 4e, Additional file 3: Fig. S12e). These results suggested immunotherapy might be suitable for cluster1 with high CD8 T cell infiltration despite T cell dysfunction.

### Support vector machine (SVM) model based on multi-omics signatures for recognizing of immune subtypes

According to the above analysis results, cluster1 was quite different with cluster2 and cluster3 in immune-related molecular and genomic characteristics, while cluster2 and cluster3 showed similar features despite the significantly different overall survival. And GSEA analysis showed DNA damage repair and inflammation pathways were enriched in cluster1, while cluster3 had significant enrichments in some metabolic pathways (Additional file 2: Table S7, Additional file 3: Fig. S13a, b).

**Fig. 5:**
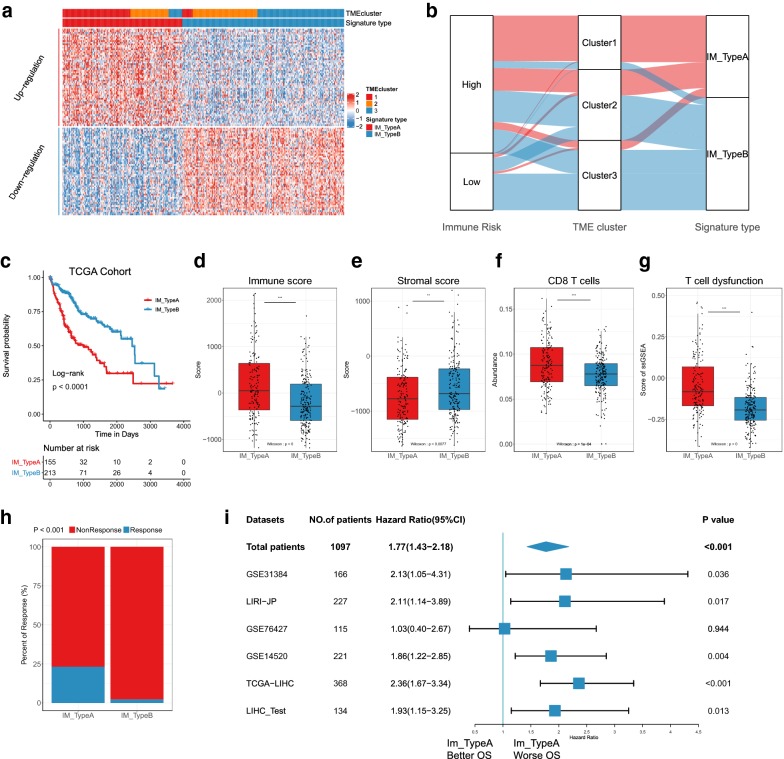
Multi-omics signatures for recognizing of immune subtypes. **a** Heatmap for the featured mRNA (by random forest selecting) distribution in SVM classifier and three clusters. Heatmap for the CpG methylation sites, LncRNAs, miRNAs and proteins can be obtained in Fig. S14A-D. **b** Visualization with sankey diagram for the distribution alteration of patients in immunoscore groups, 3 clusters and SVM subtypes. **c** Kaplan–Meier OS curves grouped by SVM classifier in TCGA cohort. Comparison of immune scores (**d**), stromal scores (**e**), CD8 T cells (**f**), T cell dysfunction (**g**) (Wilcoxon signed rank test) and predicted response to immunotherapy (**h**) (Chi-squared test) between Type A and Type B based on SVM model in TCGA cohort. **i** Forest diagram for subtype analysis between Type A and Type B in independent cohorts and whole meta HCC dataset. ns: no significance, *P < 0.05, **P < 0.01, ***P < 0.001

Next, we obtained differentially expressed proteins, mRNA, miRNA, LncRNA and CpG methylation sites. Interestingly, the differentially expressed genes between cluster1 and cluster3 were the most, while those between cluster2 and cluster3 were the least. The same results were found in miRNA, LncRNA and CpG methylation sites (Additional file 3: Fig. S13c–f). These results are consistent with the conclusions above that there was great heterogeneity between cluster1 and cluster2 or between cluster1 and cluster3. To identify the signatures of high immune risk, immune escape and better response to immunotherapies (anti-PD1/CTLA4) in HCC, we selected cluster1 and cluster3 as the phenotypes for comparison, because of the variances in clinical, molecular and genomic characteristics of cluster1 and cluster3 as described above. Boruta method [46] based on random forest algorithm was used for feature selection by dimension reduction to obtain differential mRNAs, miRNAs, DNA methylation sites and proteins, which were multi-omics signatures based on immunophenotype of HCC. Finally, 112 mRNAs, 27 miRNAs, 44 LncRNAs, 96 CpG methylation sites and 9 proteins were confirmed in determining immune subtypes (Fig. 5a, Additional file 2: Tables S8–S12, Additional file 3: Fig. S14a–d).

Then we established an SVM classifier with fivefold cross-validation based on cluster1 and cluster3. Cluster2 was set as an internal validation set since it had similar survival to cluster 1 but similar molecular and genomic characteristics to cluster 3, and GSE14520, GSE76427, LIRI-JP, microRNA dataset GSE31384 and meta-validation cohort were set external validation sets. In the training group, most patients cluster1 were trained to a group named “Type A” and patients in cluster3 were trained to “Type B” group (Fig. 5a, b). Further, we used this classifier to test the internal group cluster2 and found it could be divided into two groups and there existed survival differences between the two groups (Additional file 3: Fig. S15a), suggesting internal immune diversities in cluster2. Therefore, the TCGA cohort could be classified into two groups: Type A had worse survival, higher immune scores, lower stroma scores, higher CD8 T cells, but higher potential of immune escape, more T cell dysfunction and better response to immunotherapy than Type B (predicted immunotherapy response rate: 37/159 (23.3%) vs. 5/215 (2.3%), Chi-squared test, P < 0.001, Fig. 5c–h).

To validate the robustness of the SVM classifier, we test the model in other independent datasets (GSE14520, GSE76427, LIRI-JP and GSE31384 were used to validate the survival and meta-validation cohort were used to validate the immune characteristics). Except that patients in GSE76427 cohort, Type A had worse survival in the three external validating datasets and whole HCC datasets (Fig. 5i, Additional file 2: Table S13, Additional file 3: Fig. S15b–f). And also, Type A had higher immune scores, lower stroma scores, higher CD8 T cells, more T cell dysfunction and better response to immunotherapy (predicted immunotherapy response rate: 10/92 (10.9%) vs. 1/129 (0.1%), Fisher’s exact test, P < 0.001, Additional file 3: Fig. S15g–k). Finally, we used the SVM model to evaluate the prognosis of TCGA Pan-cancer datasets (32 types of cancer including 9783 tumors). Overall, the prognosis of each tumor type was diverse although Type A still had worse survival in pan-cancer datasets (Additional file 3: Fig. S16). In brain lower grade glioma (LGG), adrenocortical carcinoma (ACC), kidney renal clear cell carcinoma (KIRC) and pancreatic adenocarcinoma (PAAD), Type A had worse clinical outcome, while Type A had better prognosis in lung squamous cell carcinoma (LUSC), breast invasive carcinoma (BRCA) and rectum adenocarcinoma (READ) (Additional file 3: Fig. S16), implying that these tumors with similar outcome in Type A had analogous molecular characteristics, which was consistent with the results that LUSC and KIRC showed two diverse immune escape tactics and KIRC had more T cell dysfunction despite high T cell infiltration [45].

### MMP9 was a potential indicator of HCC immune characteristics

In order to further verify the validity of our multi-omics signature, we sorted all multi-omics signatures according to the P value and identified the biomarker with the lowest P value, MMP9 mRNA (Additional file 3: Fig. S17a). MMP9, a member of matrix metalloproteinase family and mainly secreted by TAM, has been to break down the extracellular matrix, inhibit interferon receptor 1, facilitate HBV DNA replication, and promote the occurrence and metastasis of HCC [47–49]. And also, inhibition of MMP9 could modulate immunosuppression in tumor [50, 51].

**Fig. 6:**
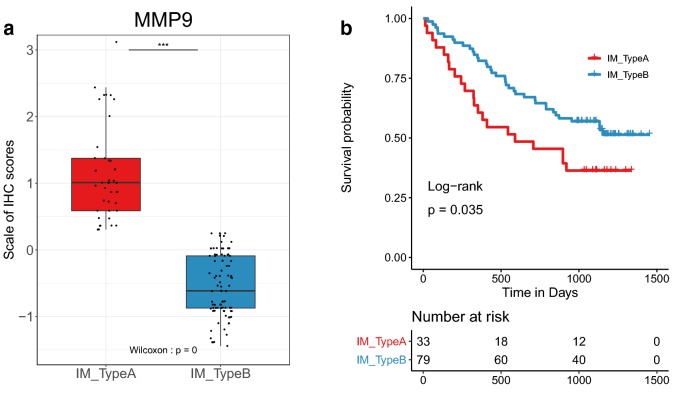
MMP9 was a potential indicator of HCC immune characteristics. **a** Comparison of MMP9 (Wilcoxon signed rank test) between Type A and Type B divided by SVM classifier based on normalized immunohistochemical staining scores of MMP9 in Tongji cohort. **b** Survival analysis of Type A and Type B based on the SVM model in Tongji cohort. ns: no significance, *P < 0.05, **P < 0.01, ***P < 0.001

Next, we performed immunohistochemical experiments to validate the relationship between MMP9 and immune characteristics in HCC. We used single-gene MMP9 to construct an SVM model based on TCGA-LIHC dataset and verified the SVM classifier in the Tongji cohort under immunohistochemical staining and scoring. In our cohort including totally 134 HCC patients, 31.3% (42/134) of the patients who were classified as Type A had higher levels of MMP9 expression, but also higher expressions of CD8A, PD1 and CTLA4. 68.7% (92/134) of the patients were Type B, which had lower expression of MMP9, CD8A, PD1, and CTLA4 than type A (P < 0.05, Fig. 6a, Additional file 3: Fig. S17b–e). In addition, we also found worse overall survival in Type A (HR = 1.77, P = 0.035, Fig. 6b). Obviously, these consistent results could be found in the TCGA-LIHC dataset, with high levels of MMP9, CD8A, PD1, and CTLA4, and poor survival in Type A (P < 0.05, HR = 1.54, Additional file 3: Fig. S18a–e). In addition, Type A with high MMP9 expression might be more suitable for immunotherapy (Additional file 3: Fig. S18f). These results showed Type A and Type B could be recognized by MMP9 at both proteins and mRNA levels, and Type A might have more CD8 T cell infiltration, accompanied by functional exhaustion caused by high expression of immune checkpoints.

## Discussion

Immunotherapy has turned into one of the most promising treatments in cancer [7, 8, 13]. However, the future of immune checkpoint therapy in liver cancer still remains unclear, with the failure of phase III clinical trial [52] (anti-PD-1) despite a small proportion (15%) of response to PD-1 inhibitor in phase II clinical trial [53]. One of the keys of immunotherapy to HCC is deeply understanding the immunological characteristics of liver cancer. In this study, we recognized the TME in HCC including differences of immune microenvironment between carcinoma and adjacent tissues, clinical, molecular and genomic characteristics in HCC immune subtypes, and signatures helping identify patients with high T cell infiltration but T cell dysfunction and higher response to immune checkpoint therapy from TCGA-LIHC and other independent cohorts.

Our work showed that most TME cells varied between tumor and adjacent non-tumor tissues. However, few researches focused on the infiltration alterations of TME cells, and we inferred that changes of immune cell composition might initiate the occurrence of tumors during the process of immune surveillance, especially in HBV and HCV related HCC. And also, the roles of TME cells in the diagnosis and prediction of liver cancer are rarely reported. This result could provide more new sights to seek the mechanisms of HCC initiation. And this research was dedicated to exploring the heterogeneity of immune subtypes of HCC based on large public datasets. There existed 3 clusters in HCC by unsupervised learning, with distinct immune cell abundance and different from the discovery of Yutaka et al. [54] that HCC patients could be purely classified as high, middle and low immune cell infiltration. The main reasons for the deviations in analysis may be the differences in immune cell estimation and classification methods, which were realized based on unsupervised clustering of machine learning in this research. Despite the distribution diversities of TME cells among the three clusters, we found cluster1 had more mature adaptive immune cells such as CD8 T cells and cytotoxic lymphocytes, which used to be considered an indicator of improved survival for cancer patients [30, 55]. Also, this conclusion was also validated in our study that high infiltration of CD8 T cells and cytotoxic lymphocytes predicted better outcome both in the whole sample and in each subtype of HCC by univariate Cox regression (Fig. 2a, b). However, cluster1 had poor survival, which may be caused by high expression of some classic or newly discovered immune checkpoints (PD1, CTLA4 and TIM-3), more infiltration of immunosuppressive cells (TAMs, Tregs and Th17 cells), and genomic alterations (TP53 mutation and deletion). Meanwhile, there are some anti-tumor characteristics in cluster1, such as high lymphocyte infiltration of CD8 T cell and lymphocyte, high IFN-γ response, and high expression of immune co-stimulators, which indicated that there may be more immunotherapeutic responses [40, 56]. Furthermore, we also found that cluster1 might be more suitable for immunotherapy, which is consistent with the high expression of some immunocheckpoints and makes up for the lack of differences in the expression of classic checkpoints such as PD-L1. However, this conclusion will need to be confirmed by clinical trials in the future.

What’s more, it is worth reminding that our results did not contradict previous findings that high infiltration of CD8 T cells indicated beneficial prognosis, but extended and enriched this conclusion. We demonstrated that there is a group of HCC patients with higher CD8 T cell infiltration, but T cell dysfunction and increased immune escape, resulting in a poor prognosis, which was consistent with discoveries in other tumors [43, 57]. These results, to some extent, explain the unsatisfactory situation of immunotherapeutic response in HCC [52, 53, 58], which are in concordance with our results. It further indicates that some patients with increased T cell infiltration are more likely to receive immunotherapy, but show not very high responsiveness for the increased immune escape and T cell dysfunction.

To recognize and immune subtype and predict the response of immunotherapy in HCC, multi-omics signatures were obtained. Using SVM classifier, we regrouped HCC patients into two groups and Type A showed similar characteristics (clinical outcomes, CD8 T cell, T cell dysfunction and response to immunotherapy) with cluster1 in both TCGA cohort and external validation cohorts. Due to the limited responsiveness of targeting immune checkpoint therapy, only a small part of patients show response. The construction of multi-omics SVM model in our study could maximally predict the benefit of immunotherapy in patients with HCC. Additionally, the SVM model could also predict prognosis of several other cancers, suggesting that these tumors might have similar or opposite immune mechanisms to HCC, which was consistent with previous studies that READ, LUSC and BRCA belonged to C1 and C2, while LIHC, PAAD, ACC, LGG belonged to C3,C4 and C5 type [59–61]. These results imply the multi-omics signatures could provide new clues to investigate the TME of HCC or other tumors in future.

Furthermore, we preliminarily verified the immune role of MMP9, a secreted protein produced by TAMs [47], in HCC through immunohistochemical experiments. High expression of MMP9 indicated higher levels of PD1, CTLA4 and CD8A and poor survival in partial HCC patients, which was in line with our above analysis that some HCC patients with high CD8 T cell infiltration but dysfunction were immunosuppressed. MMP9 can affect the immune state through a variety of ways, such as releasing VEGF to promote angiogenesis [62] and binding CD44 to release TGFβ [63]. Furthermore, MMP9 can be used as a biomarker of chemotherapy response, with high expression of MMP9 meaning better responsiveness to chemotherapy [64]. Perhaps, MMP9 could be a good indicator of T cell dysfunction and immunotherapy responsiveness. In addition to anti-PD1/CTLA4 immune checkpoint therapy, our study suggests that the combination of anti-checkpoint with anti-MMP9 [51] or anti-TAMs [65] may be more beneficial to patients with T cell dysfunction in HCC. However, due to the lack of large sequenced HCC cohort and prospective clinical trials that have received immunotherapy, the effect of MMP9 expression on the efficacy of immunotherapy in HCC patients remains concerned.

The advantage of our study is that we used a large number of publicly available independent data sets (from TCGA, ICGC and GEO) and our own cohort, applying different research methods (genomics, transcriptomics, IHC and so on) to detect problems and verify them, which makes the conclusions consistent and reproducible. However, our study is still limited in that the data sources we used were all retrospective. In addition, most of our conclusions are based on bioinformatics analysis. Although multiple datasets from different sources show feasibility, it still needs further experimental verification and application. And the intriguing perspectives and conjectures on our multi-omics SVM model and the immunological role of MMP9 in this study need to be further verified in future prospective clinical trials and molecular biology researches.

Overall, in this research, comprehensive analysis and assessment of TME patterns based on multi-omics in HCC can provide some new strategies about response to immunotherapy, and the combination of targeting drugs.

## Conclusions

Our work demonstrated 3 immune clusters with different features. More importantly, multi-omics signatures, such as MMP9 was identified based on three clusters to help us recognize patients with different prognosis and responses to immunotherapy in HCC. This study could further reveal the immune status of HCC and provide potential predictors for immune checkpoint treatment response.

## Supplementary information

**Additional file 1.** Additional materials and months.

**Additional file 2.** Additional tables.

**Additional file 3.** Additional figures.

## Data Availability

All data generated or analyzed during this study are included in the manuscript and its additional files.

## Abbreviations

HCC: Hepatocellular carcinoma
TAM: Tumor-associated macrophage
MDSC: Myeloid-derived suppressor cell
TME: Tumor microenvironment
LncRNA: Long non-coding RNA
TCGA-LIHC: The Cancer Genome Atlas-Liver hepatocellular carcinoma (TCGA-LIHC)
ICGC: The International Cancer Genome Consortium
AFP: α-Fetoprotein
TMB: Tumor mutation burden
CNV: Copy number variation
ITH: Intratumoral heterogeneity
HRD: Homologous recombination deficiency
LOH: Loss of heterozygosity
OS: Overall survival
SVM: Support vector machine
