# Supplementary material for: Microenvironment characterization and multi-omics signatures related to prognosis and immunotherapy response of hepatocellular carcinoma
Source: Exp Hematol Oncol. 2020 May 25;9:10. doi: 10.1186/s40164-020-00165-3 (PMC7249423; doi:10.1186/s40164-020-00165-3)
Supplement: Supplementary file 1 — Additional file 1. Additional materials and months. [file 40164_2020_165_MOESM1_ESM.docx]

# Additional materials and methods：

## Datasets and collection:

**GEO datasets (**[**https://www.ncbi.nlm.nih.gov/gds**](https://www.ncbi.nlm.nih.gov/gds)**).** In total, we gathered six cohorts from, including: GSE63898 (Platform: GPL13667, Affymetrix Human Genome U219 Array, 228 HCC samples and 168 adjacent tissues), GSE76297 (Platforms: GPL17586, Affymetrix Human Transcriptome Array 2.0, 62 HCC samples and 59 adjacent tissues), GSE76427 (Platforms: GPL10558, Illumina HumanHT-12 V4.0 expression beadchip, 115 HCC samples and 52 adjacent tissues), GSE64041 (Platforms: GPL6244, Affymetrix Human Gene 1.0 ST Array, 60 HCC samples and 60 adjacent tissues), GSE14520 (Platforms: GPL571, Affymetrix Human Genome U133A 2.0 Array), GSE31384 (Platforms: GPL14140, CapitalBio custom Human microRNA array). Each probe was annotated with the platform. For the repetitive probe, the one of maximum expression was retained. And clinical data of GSE76427, GSE14520, GSE31384 was obtained from their metadata or supplementary files.

**ICGC datasets(**[**https://icgc.org/**](https://icgc.org/)**).** Two RNA-Seq datasets were obtained from ICGC. LICA-FR contained gene expression profile of 161 French HCC patients. LIRI-JP, a Japanese HCC dataset, had 232 HCC patients with clinical outcome data. All normalized read counts were downloaded. For LICA-FR, gene symbol was annotated with the GRCh38.98.chr.gtf file downloaded from Ensembl (<ftp://ftp.ensembl.org/pub/release-98/gtf/homo_sapiens>)

**TCGA-LIHC.** For HCC datasets LIHC, Level 4 data was downloaded from xenahub GDC TCGA Liver Cancer (<https://xenabrowser.net/datapages/>), including RNA-seq gene expression count data, Copy Number Segment(hg38), somatic mutation maf file(hg38, MuTect2 Variant Aggregation and Masking), miRNA expression datasets, Illumina Human Methylation 450 (hg38). TCGA-LIHC clinical information was downloaded from xenahub (up to 07-20-2019). Gene symbol was annotated with the GRCh38.98.chr.gtf file downloaded from Ensembl. Long non-coding RNA and mRNA was separated by the gene type in the gene annotation procession. For miRNAs and lncRNAs, RNA with more than 20% zero values among the all HCC samples was removed. And Copy number data was processed with the Gistic2.0. Gene Mapping for CpG islands sites was performed with the illuminaMethyl450_hg38_GDC file, and CpG islands site with more than 20% missing values was removed. Reverse phase protein lysate microarray (RPPA) including 218 canonical proteins of liver cancer was extracted from pan-cancer RPPA dataset to facilitate the protein research required for this study, and the proteins with more than 20% missing value were deleted.

**Pan-cancer dataset:** we downloaded the pan-cancer RNA-seq data and clinical outcome information (up to 2018-09-13) from xenahub.

For each expression data, we fill out the missing value with the *impute* R package. Every dataset was normalized with the *normalizeBetweenArrays* function in *limma*[1] package before analysis except for RNA-seq data during immune cell estimation in CIBERSORT as authors recommended[2].

To estimate the abundance of immune cells more accurately, we screened datasets with genes in the dataset covering more than 95% genes in leukocyte signature matrix (LM22) of CIBERSORT and immune-related genes of MCPcounter[3]. Therefore, the validation cohort (N=626) was comprised of GSE63898 (N=228), GSE76297 (N=62), GSE76297 (N=115), GSE64041 (N=60), LICA-FR (N=161). The batch effect in the meta validation cohort was eliminated by *sva*[4] package.

## Estimation of the abundance of tumor environmental cells

We used CIBERSORT and MCPcounter to compute the infiltration of 26 TME cells by using gene expression data of TCGA-LIHC and meta validation cohort. LM22 gene signature (including 567 immune related genes) and CIBERSORT method (<https://cibersort.stanford.edu/>) were runned with 1000 permutations to quantify 22 TME cells. MCPcounter method based on R package *MCPcounter* was performed to quantify 10 TME cells (8 immune cells and 2 stroma cells) robustly. Finally, 26 immune cells include myeloid dendritic cells, fibroblasts, endothelial cells, cytotoxic lymphocytes, neutrophils calculated by MCPcounter and other 21 immune cells computed by CIBERSORT (except neutrophils). All gene expression datasets were normalized.

## Construction of Least Absolute Shrinkage and Selection Operator (LASSO) prognostic model and immunoscore based on TME cells

As described in our previous study[5], to assess the prognosis of each TME cell, we performed univariate cox regression analysis on each TME cell and screened those with P < 0.05. Then those TME cells that significantly affected survival were comprehensively evaluated by the LASSO model, which could avoid the deviation caused by collinearity among abundances of TME cells. Also, immunoscore of each patient was calculated by *predict* function in R. LASSO model was constructed by *glmnet*[6] package in R. Immunorisk phenotype (High risk and low risk) was defined by the optimal cutoff value based on immunoscore with *surv_cutpoint* function in *survminer* package.

## Immune subtype identification in discovery and validation groups

First, expectation-maximization algorithm carried out by *mclust*[7] was applied to screen the optimal number of clusters. Then we performed unsupervised clustering to confirm the number of immune subtype and determine the subtype to which each patient belonged in both discovery and validation groups, and the optimal number of clusters was determined: (1) by the *NbClust*[8] package, which provided 30 indexes to confirm the cluster number. (2) by the area of empirical cumulative distribution function of consensus matrix at each cluster. Unsupervised clustering was carried out by *NbClust* and *ConsensusClusterPlus*[9] packages, and parameters were set as K-means method, euclidean distance and 1000 repeats to confirm the stable results.

The distribution consistency of TME cells in each subtype of discovery and validation group was evaluated. In each immune subtype both in discovery and validation cohorts, the centroid of each patient's immune cells was calculated. And then linear correlation between centroids in different clusters of discovery and validation cohorts were calculated by Pearson correlation.

## Evaluation of immune related molecular characteristics and genomic alterations

We evaluated 26 molecular features or oncogenic pathways scores among the immune subtypes, including: CD8 T cell effector, Th1, Th2 and Th17 cells, IFNγ response, reactive stroma, angiogenesis, TNFα response, hepatic fibrosis, differentiation, DNA damage repair (DDR) response, wound healing, proliferation, leukocyte fraction, HBV counts, HCV counts, indel, immunogenic mutation, immunogenic indel, CNV burden, intratumoral heterogeneity (ITH), homologous recombination deficiency (HRD), loss of heterozygosity (LOH) and aneuploidy. We got the CD8 T cell effector gene set from Jonathan E et al.[10]. DNA damage repair (DDR) response gene sets came from Lange SS et al.[11]. For the Th1, Th2 and Th17 cells, IFNγ response, reactive stroma, angiogenesis, TNFα response, hepatic fibrosis, differentiation, wound healing, proliferation, we obtained the gene sets from 3 literatures[12-14] and a public database (<https://immport.niaid.nih.gov/home>)[15] and then took the intersection of the common parts of three data sets. For these gene sets, we used single-sample gene set enrichment (ssGSEA) method based on *GSVA* package[16] to evaluate the overall level of single gene set including multiple genes. Leukocyte fraction, estimated by a mixture model based on the most differentiated methylation sites between the most differentiated methylation sites between white blood cells and normal tissue and normal tissues, could be downloaded from Pan-cancer immune[12], where HBV and HCV counts, indel, immunogenic mutation, immunogenic indel, CNV burden, ITH, HRD, LOH and aneuploidy scores could be also obtained.

## Mutation and copy number variations (CNVs) analysis

Somatic mutation maf file (hg38, MuTect2 Variant Aggregation and Masking) and Copy Number Segment(hg38) were downloaded as above described. We removed the silent mutation in all samples. Tumor mutation burden (TMB), a potential biomarker for immunotherapy responsiveness despite existing some controversies, was defined and calculated as the number of mutations per Mb in the genome for each patient. All visualizations of analysis related mutation were performed by *maftools*[17]. Specific mutations in each subtype were compared with Chi-squared test or Fisher's exact test. CNVs of each immune subtype were performed in GISTIC 2.0 (<https://cloud.genepattern.org/>) with default parameters, and specific amplification and deletion regions and genes were compared with Chi-squared test or Fisher's exact test.

## Gene Set Enrichment Analysis

We used *clusterprofiler* package[18] to complete Gene Set Enrichment Analysis (GSEA). Hallmark gene sets v7.0 was selected as Molecular Signatures Database.

## Immunomodulators analysis among immune subtypes

We compared three studies[12, 19, 20] and identified 76 immunomodulators (14 antigen presentation molecules, 23 inhibitors and 37 stimulators; Additional file 2: TableS7). And we analyzed the immunomodulators’ gene expressions and compared them in the three clusters with Kruskal–Wallis test. For the immunomodulators with significance (P < 0.05), we analyzed the DNA methylation of their promoters including the beta values of the probes and the correlation between immunomodulator expressions and their corresponding probes (Spearman’s correlations), and CNVs including amplification and deletion. Also, the Spearman’s correlations between miRNA and their targeted immunomodulator expressions. The predicted corresponding miRNAs of the genes were obtained from miRDB database (<http://www.mirdb.org/>)[21].

## Identification of multi-omics signatures associated with immune subtypes

To seek signatures to understand the patterns of TME cell infiltration and recognize immune subtypes, multi-omics signatures (mRNA, long non-coding RNA, miRNA, CpG methylation sites and proteins) were prepared to differentially analyzed. Kruskal–Wallis test was used to identify significant proteins from RPPA, proteins with P value less than 0.05 were considered significant. For DNA methylation data, beta values were converted to M values by function *beta2m* in *lumi* package[22]. Next, mRNA, LncRNA with log_2_(counts+1) and DNA methylation with M values were differentially analyzed by *limma* package with an empirical Bayesian algorithm (|Log_2_FC| > 1, P < 0.05 and FDR < 0.1), and miRNA with log_2_(RPM+1) was screened by *limma* package with the threshold value, |Log_2_FC| > 0.5, P < 0.05 and FDR < 0.1. For these differential multi-omics signatures, we performed Boruta feature selection algorithm based on random forest classification to reduce dimension[23]. Finally, we screened signatures strong correlated with immunotype and discarded redundant markers.

## Support vector machine (SVM) classifier recognizing immunotypes

After obtaining the multi-omics signatures, in order to use these signatures data for analysis and prediction of immunotypes, we used the supervised clustering method based on support vector machine (SVM) to classify the training and testing dataset. Considering the immune risk score, survival, immune characteristics, genomic characteristics of each cluster, as well as the predicted immunotherapeutic response, we took cluster1 and cluster3 as the classification outcomes for supervised learning, and obtained Type A and Type B. An SVM classifier based on each omics data screened by Boruta was established in TCGA-LIHC dataset and validated in other external datasets (including mRNA and miRNA groups) with the common features. Both training and testing datasets were standardized in each omics group as previous described[24], and then normalized training omics profiles were trained as immune-related SVM classifier to find optimal hyperparameters with 5-fold cross validation. Finally, we applied the SVM model to each sample from testing datasets to classify the patients into related immunotype. SVM model was trained and tested with *e1071* package.

## Estimating immune score, stromal score, T cell dysfunction and immunotherapy responsiveness

Immune score and stromal score, indicators of overall immune cell and stroma cell infiltration, calculated by *ESTIMATE* algorithm[25]. T cell dysfunction scores were calculated by ssGSEA with defined gene set (TGFB1, CD274, CTLA4, IL10, PDCD1, CD276, HAVCR2, TNFRSF9, LAG3, TIGIT, ICOS), in which genes negatively regulate T cell function. And we performed Tumor Immune Dysfunction and Exclusion (TIDE, <http://tide.dfci.harvard.edu/>) tool[26] to estimate the clinical responsiveness to immune checkpoint blockade (anti-PD1/CTLA4). Also, we used the recommended normalized datasets to upload the website. Notably, only three datasets (discovery dataset: TCGA-LIHC; validation cohorts: GSE64041, LICA-FR) could be used to be analyzed in the tool because of the strict condition for gene number.

## Immunohistochemical (IHC) analysis

IHC assay was performed as described previously[27]. The following primary antibodies were used: MMP9 (Santa-Cruz, mouse monoclonal, sc-21733, USA; 1:500 dilution), CTLA4 (Santa-Cruz, mouse monoclonal, sc-376016; 1:250 dilution), PD1 (Abcam, rabbit monoclonal, ab137132; 1:200 dilution), CD8A (Gene Tech (Shanghai), rabbit monoclonal, GT2112;1:100 dilution). For MMP9 and CTLA4 IHC staining scores, we used a semiquantitative as described previously[28] with staining intensity scores determining as follows: 0 (negative), 1 (weak), 2 (moderate), 3 (strong) and stained positive cell scores determining as follows: 0 (0%-10%), 1 (10–25%), 2 (25–50%), 3 (50–75%), 4 point (75–100%) since these two proteins were detected in tumor cells. Finally, the total staining scores were obtained by staining intensity scores × stained positive cell scores. For the other three expressed proteins specifically (PD1, CD8A), the stained specific cells were compared with the stromal nucleated cells as previously reported[29], and the scoring methods were the same as the semi-quantitative methods described above. The scoring process was conducted by two independent experienced pathologists blinded to the study. Then we normalized the IHC staining scores of each protein and MMP9 mRNA expression from TCGA. Then we constructed an SVM classifier applied the IHC patients for the following analysis.

All packages and visualizations in this study were conducted in R software (version: 3.6.1).

**References:**

1. Ritchie ME, Phipson B, Wu D, Hu Y, Law CW, Shi W, et al. limma powers differential expression analyses for RNA-sequencing and microarray studies. NUCLEIC ACIDS RES. 2015;43:e47.

2. Newman AM, Liu CL, Green MR, Gentles AJ, Feng W, Xu Y, et al. Robust enumeration of cell subsets from tissue expression profiles. NAT METHODS. 2015;12:453-7.

3. Becht E, Giraldo NA, Lacroix L, Buttard B, Elarouci N, Petitprez F, et al. Estimating the population abundance of tissue-infiltrating immune and stromal cell populations using gene expression. GENOME BIOL. 2016;17:218.

4. Leek JT, Johnson WE, Parker HS, Jaffe AE, Storey JD. The sva package for removing batch effects and other unwanted variation in high-throughput experiments. BIOINFORMATICS. 2012;28:882-3.

5. Liu F, Liao Z, Song J, Yuan C, Liu Y, Zhang H, et al. Genome-wide screening diagnostic biomarkers and the construction of prognostic model of hepatocellular carcinoma. J CELL BIOCHEM. 2019.

6. Friedman J, Hastie T, Tibshirani R. Regularization Paths for Generalized Linear Models via Coordinate Descent. J STAT SOFTW. 2010;33:1-22.

7. Scrucca L, Fop M, Murphy TB, Raftery AE. mclust 5: Clustering, Classification and Density Estimation Using Gaussian Finite Mixture Models. R J. 2016;8:289-317.

8. Charrad M, Ghazzali N, Boiteau V, Niknafs A. NbClust: An R Package for Determining the Relevant Number of Clusters in a Data Set. J STAT SOFTW. 2014;61:1-36.

9. Monti S, Tamayo P, Mesirov J, Golub T. Consensus Clustering: A Resampling-Based Method for Class Discovery and Visualization of Gene Expression Microarray Data. MACH LEARN. 2003;52:91-118.

10. Rosenberg JE, Hoffman-Censits J, Powles T, van der Heijden MS, Balar AV, Necchi A, et al. Atezolizumab in patients with locally advanced and metastatic urothelial carcinoma who have progressed following treatment with platinum-based chemotherapy: a single-arm, multicentre, phase 2 trial. LANCET. 2016;387:1909-20.

11. Lange SS, Takata K, Wood RD. DNA polymerases and cancer. NAT REV CANCER. 2011;11:96-110.

12. Thorsson V, Gibbs DL, Brown SD, Wolf D, Bortone DS, Ou YT, et al. The Immune Landscape of Cancer. IMMUNITY. 2018;48:812-30.

13. Li B, Cui Y, Nambiar DK, Sunwoo JB, Li R. The immune subtypes and landscape of squamous cell carcinoma. CLIN CANCER RES. 2019:2018-4085.

14. Chen J, Zaidi S, Rao S, Chen JS, Phan L, Farci P, et al. Analysis of Genomes and Transcriptomes of Hepatocellular Carcinomas Identifies Mutations and Gene Expression Changes in the Transforming Growth Factor-beta Pathway. GASTROENTEROLOGY. 2018;154:195-210.

15. Bhattacharya S, Andorf S, Gomes L, Dunn P, Schaefer H, Pontius J, et al. ImmPort: disseminating data to the public for the future of immunology. IMMUNOL RES. 2014;58:234-9.

16. Hanzelmann S, Castelo R, Guinney J. GSVA: gene set variation analysis for microarray and RNA-seq data. BMC BIOINFORMATICS. 2013;14:7.

17. Mayakonda A, Lin DC, Assenov Y, Plass C, Koeffler HP. Maftools: efficient and comprehensive analysis of somatic variants in cancer. GENOME RES. 2018;28:1747-56.

18. Yu G, Wang LG, Han Y, He QY. clusterProfiler: an R package for comparing biological themes among gene clusters. OMICS. 2012;16:284-7.

19. Vidotto T, Nersesian S, Graham C, Siemens DR, Koti M. DNA damage repair gene mutations and their association with tumor immune regulatory gene expression in muscle invasive bladder cancer subtypes. J IMMUNOTHER CANCER. 2019;7:148.

20. Yi M, Jiao D, Xu H, Liu Q, Zhao W, Han X, et al. Biomarkers for predicting efficacy of PD-1/PD-L1 inhibitors. MOL CANCER. 2018;17:129.

21. Liu W, Wang X. Prediction of functional microRNA targets by integrative modeling of microRNA binding and target expression data. GENOME BIOL. 2019;20:18.

22. Du P, Zhang X, Huang CC, Jafari N, Kibbe WA, Hou L, et al. Comparison of Beta-value and M-value methods for quantifying methylation levels by microarray analysis. BMC BIOINFORMATICS. 2010;11:587.

23. Kursa MB, Rudnicki WR. Feature Selection with the Boruta Package. J STAT SOFTW. 2010;36:1-13.

24. Chaudhary K, Poirion OB, Lu L, Garmire LX. Deep Learning-Based Multi-Omics Integration Robustly Predicts Survival in Liver Cancer. CLIN CANCER RES. 2018;24:1248-59.

25. Yoshihara K, Shahmoradgoli M, Martinez E, Vegesna R, Kim H, Torres-Garcia W, et al. Inferring tumour purity and stromal and immune cell admixture from expression data. NAT COMMUN. 2013;4:2612.

26. Jiang P, Gu S, Pan D, Fu J, Sahu A, Hu X, et al. Signatures of T cell dysfunction and exclusion predict cancer immunotherapy response. NAT MED. 2018;24:1550-8.

27. Tan X, Liao Z, Liang H, Chen X, Zhang B, Chu L. Upregulation of liver kinase B1 predicts poor prognosis in hepatocellular carcinoma. INT J ONCOL. 2018;53:1913-26.

28. Chen J, Zhu H, Liu Q, Ning D, Zhang Z, Zhang L, et al. DEPTOR induces a partial epithelial-to-mesenchymal transition and metastasis via autocrine TGFbeta1 signaling and is associated with poor prognosis in hepatocellular carcinoma. J Exp Clin Cancer Res. 2019;38:273.

29. Dong ZY, Zhong WZ, Zhang XC, Su J, Xie Z, Liu SY, et al. Potential Predictive Value of TP53 and KRAS Mutation Status for Response to PD-1 Blockade Immunotherapy in Lung Adenocarcinoma. CLIN CANCER RES. 2017;23:3012-24.
