# Supplementary material for: Microenvironment characterization and multi-omics signatures related to prognosis and immunotherapy response of hepatocellular carcinoma
Source: Exp Hematol Oncol. 2020 May 25;9:10. doi: 10.1186/s40164-020-00165-3 (PMC7249423; doi:10.1186/s40164-020-00165-3)
Supplement: Supplementary file 3 — Additional file 3. Additional figures. [file 40164_2020_165_MOESM3_ESM.pdf]

Additional Figures:

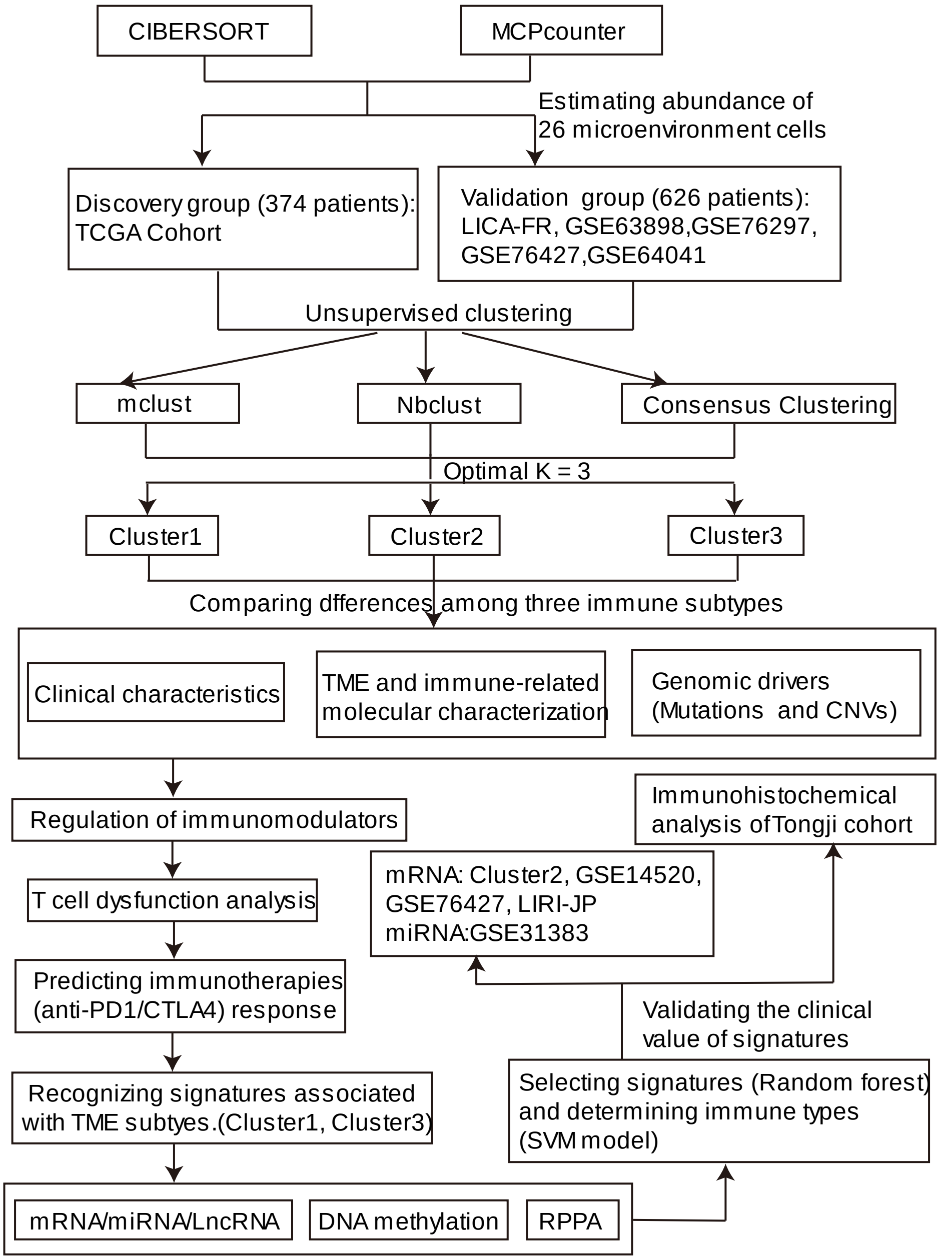

Fig. S1. The workflow of this study.

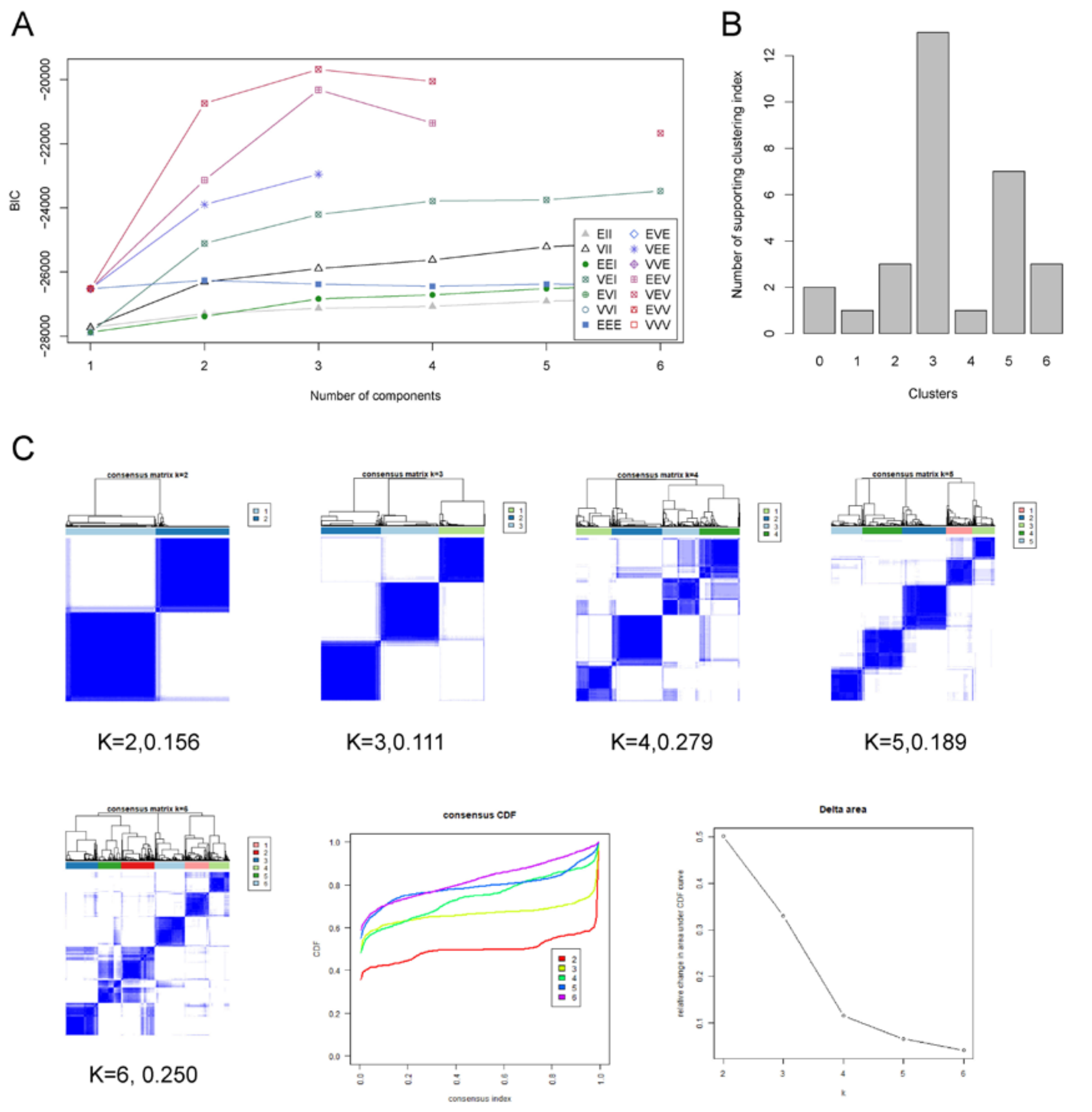

Fig. S2. Three clustering methods are used to identify the optimal number of clusters in TCGA-LIHC. A mclust. **B** NbClust. **C** ConsensusClusterPlus, the number in each consensus matrixes meaned the errors in different K.

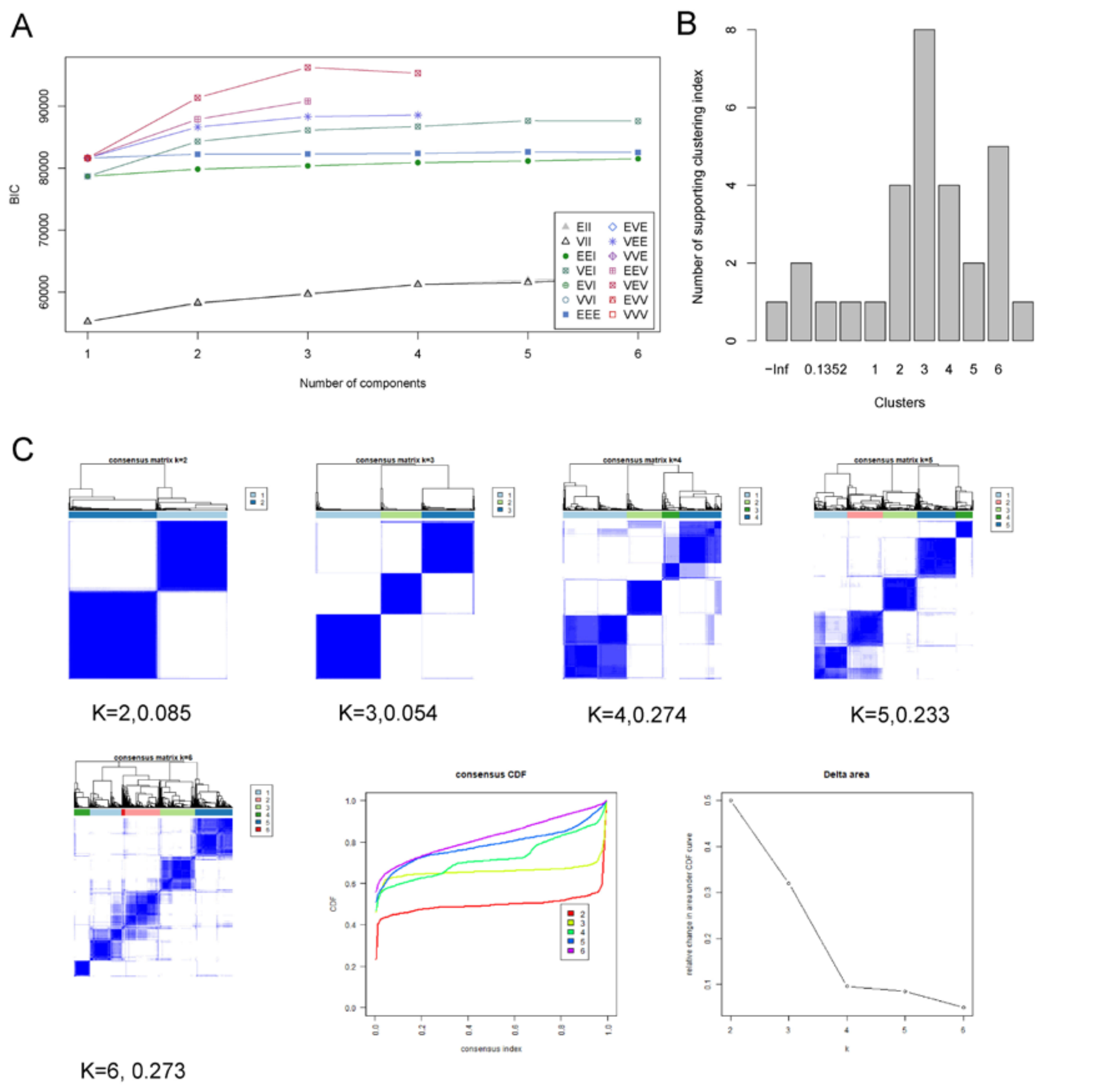

Fig. S3. Three clustering methods are used to identify the optimal number of clusters in the meta-validation cohort. A mclust. **B** NbClust. **C** ConsensusClusterPlus, the number in each consensus matrixes meaned the errors in different K.

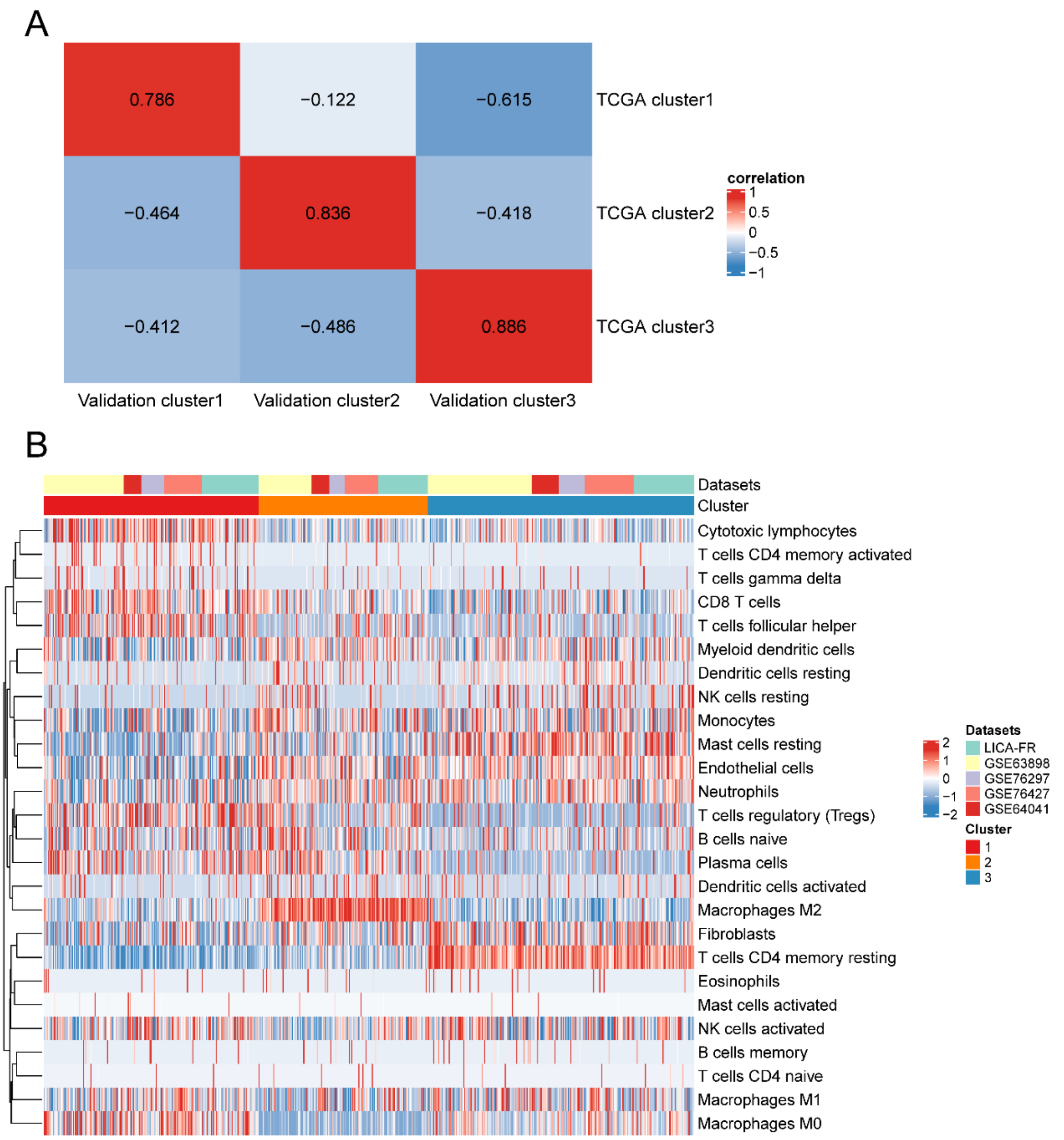

Fig. S4. The consistency of clustering between LIHC and meta-validation cohort and distribution of TME cells in validation cohort. A The Pearson correlation of each cluster in TCGA-LIHC and meta-validation cohort. **B** Heatmap of distribution of TME cells in validation cohort among three clusters.

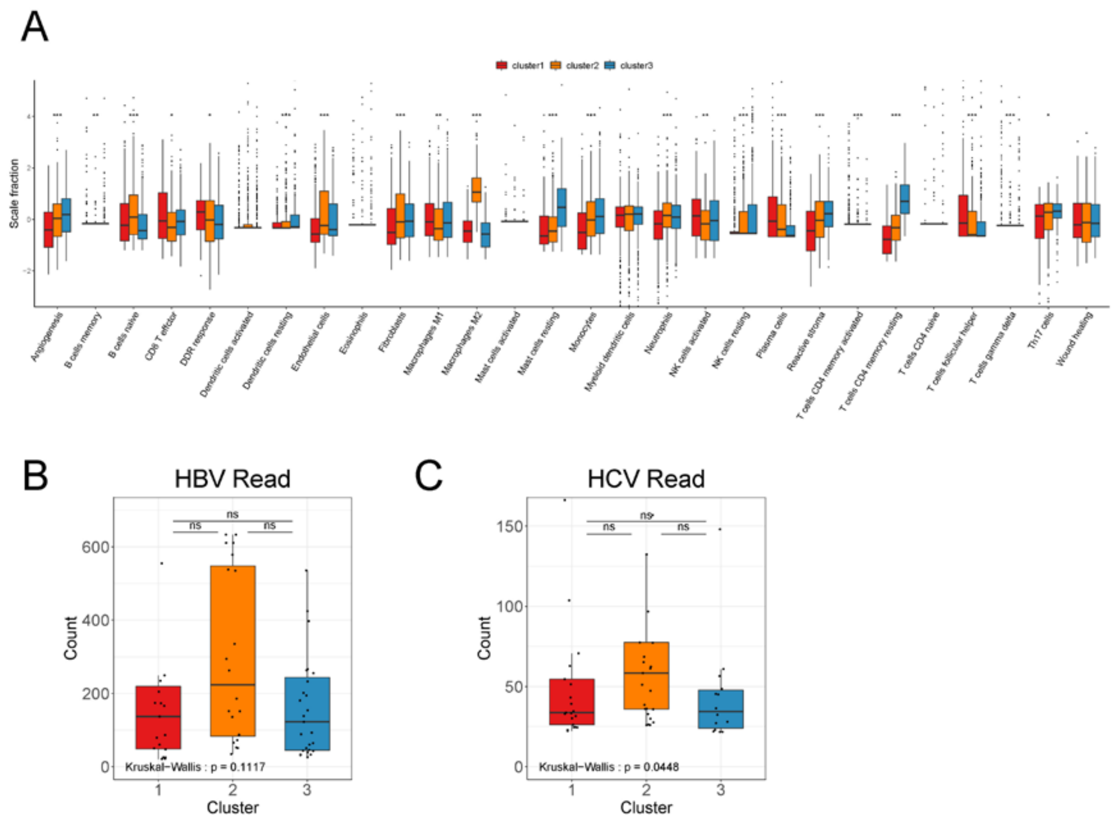

Fig. S5. Molecular characteristics among three immune subtypes. Comparison of other immune cells and biological processes (**A**), HBV and HCV DNA reads (**B**), (**C**).

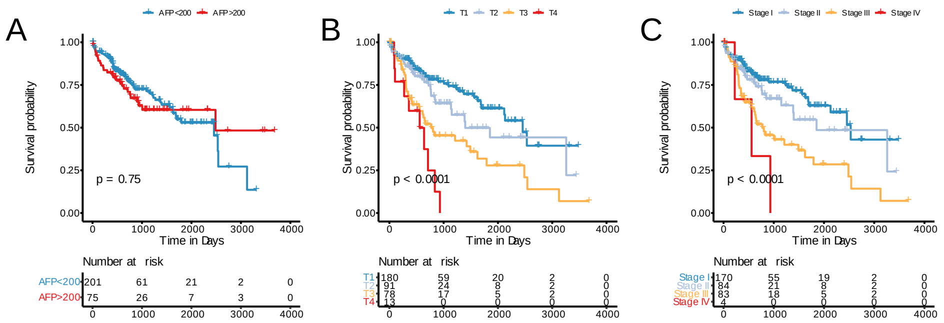

Fig. S6. Survival analysis of AFP (A), T stage (B) and pathological stage (C).

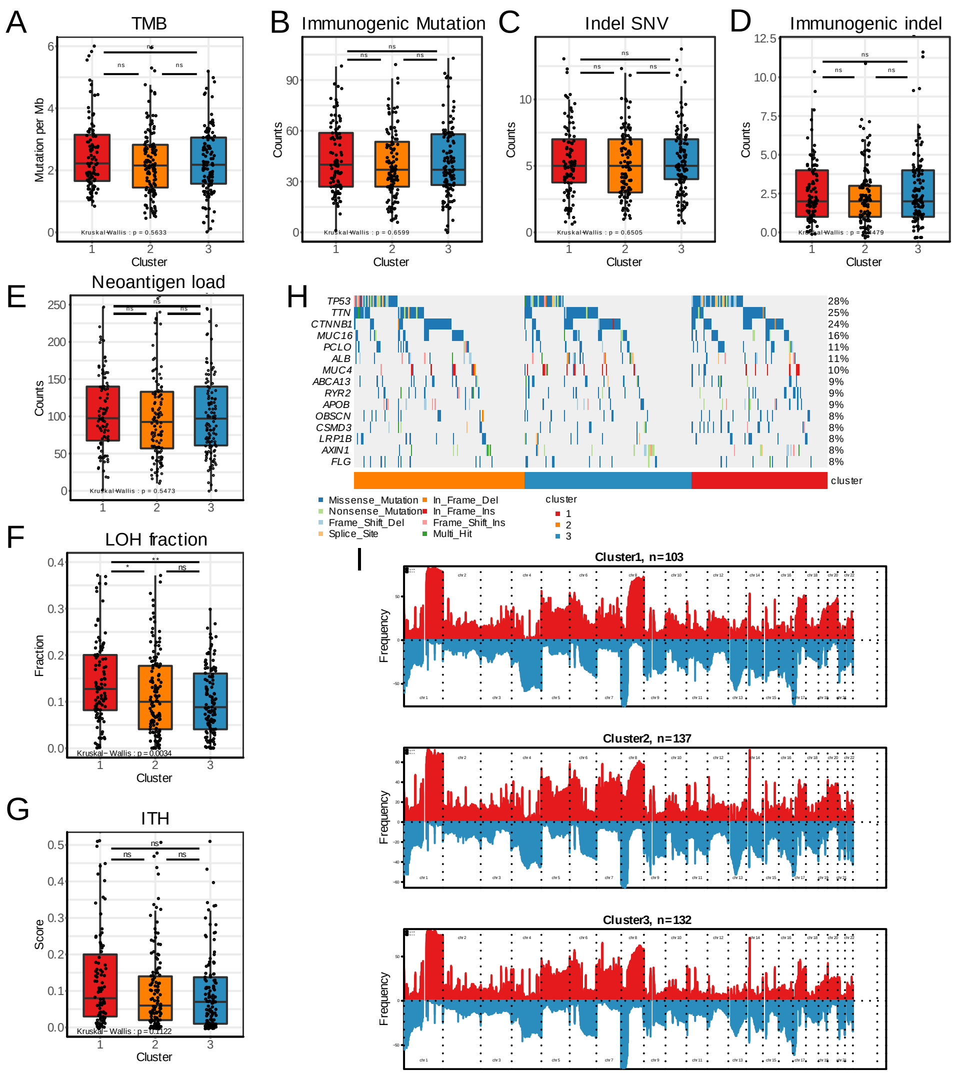

Fig. S7. The immunogenicity and mutation alterations among three clusters. Comparison of TMB (**A**), immunogenic mutations (**B**), indel SNVs (**C**), immunogenic indels (**D**), Neoantigen load (**E**), LOH fractions **(F)** and ITH scores **(G)** among three clusters. **H** Oncoprint map of 15 most mutated genes. **I** Frequency of CNV among three clusters. Red: Gain/Amplification; Blue: Loss/Deletion. Heatmap of significant deletion genes in each subtype (Chi-squared test or Fisher's exact test, FDR < 0.1).

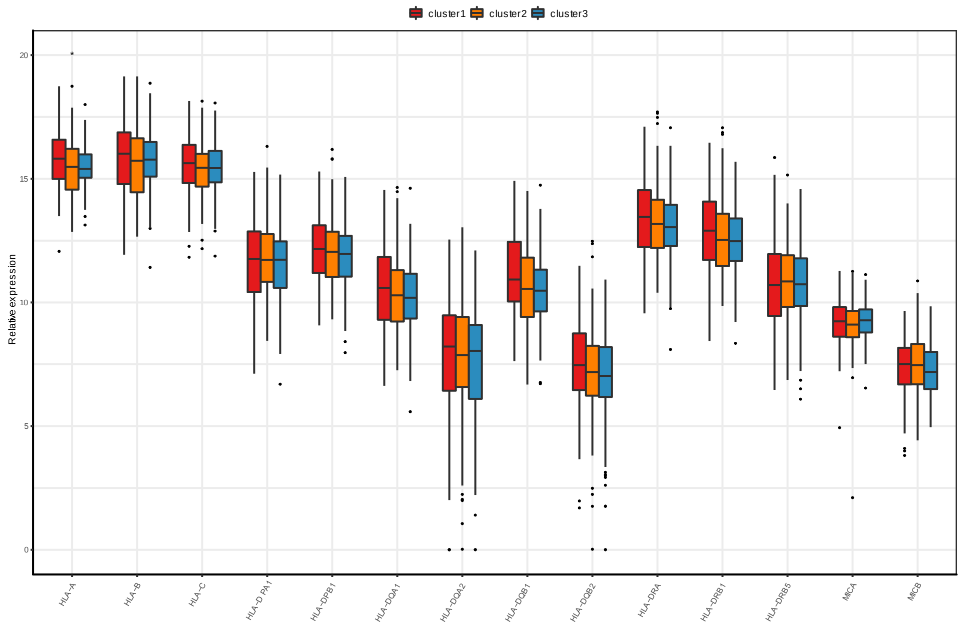

Fig. S8. The mRNA expression of antigen presentation molecules.

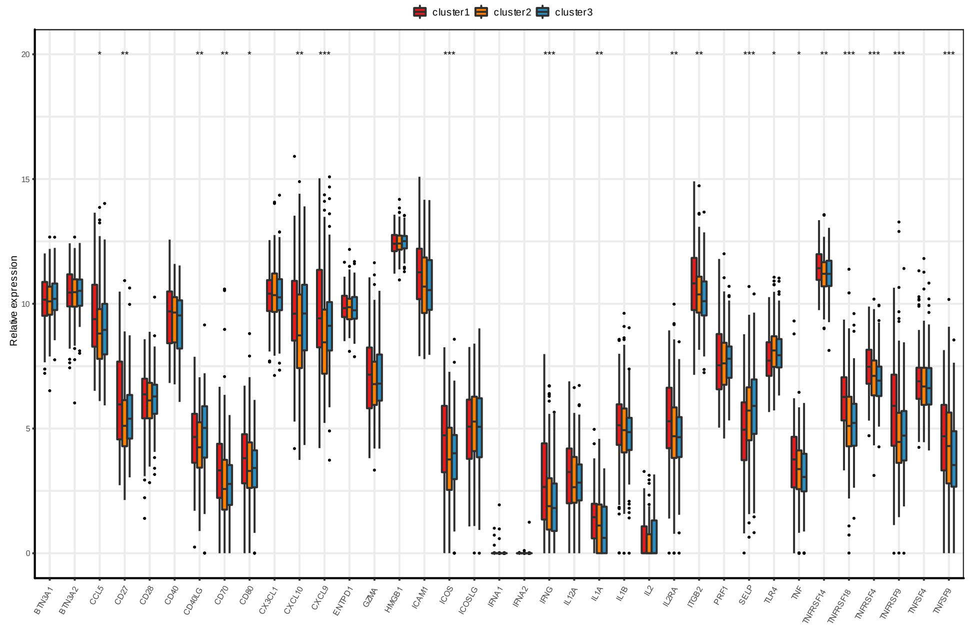

Fig. S9. The mRNA expression of immunological stimulators.

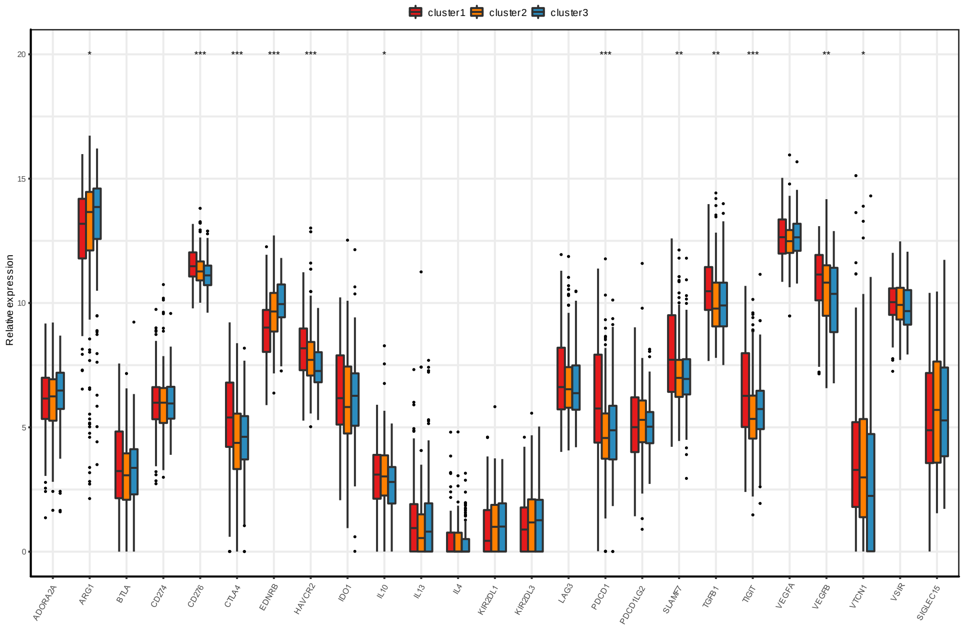

Fig. S10. The mRNA expression of immunological inhibitors.

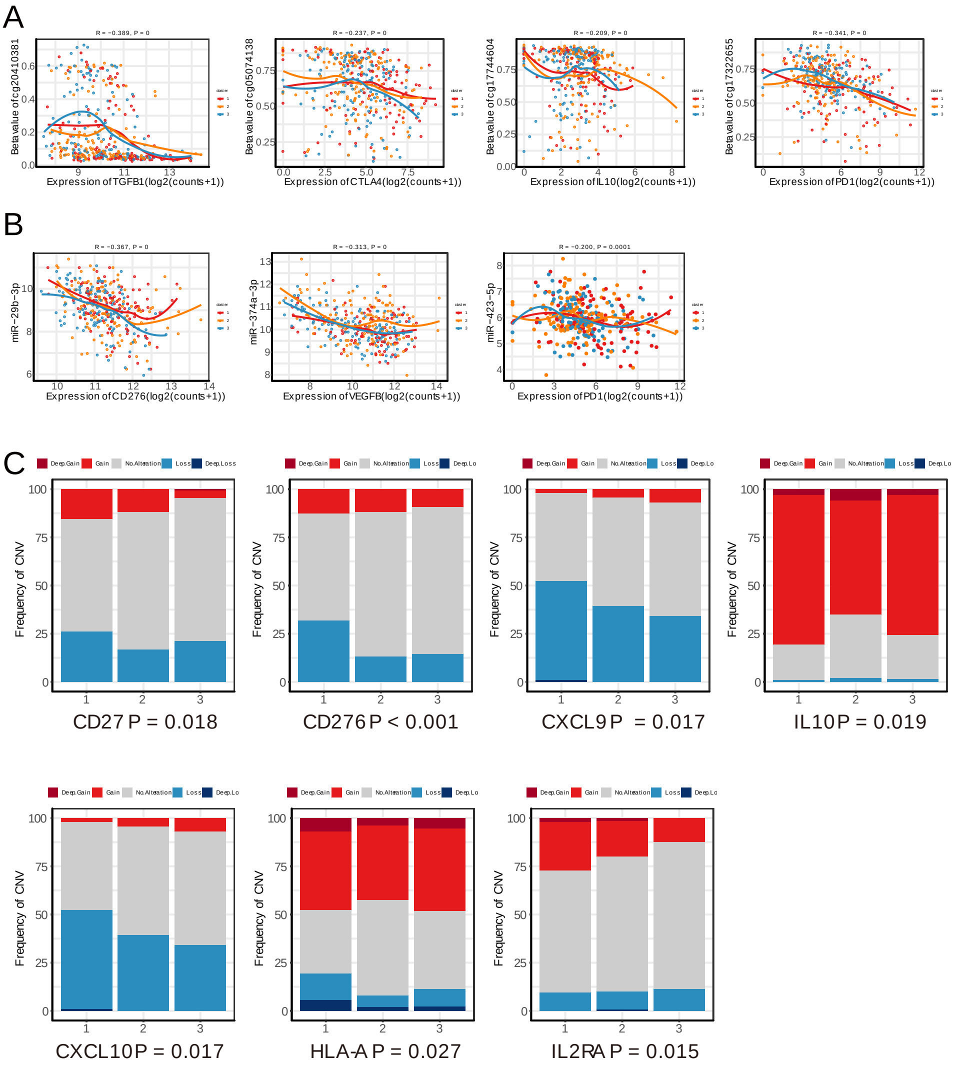

Fig. S11. Regulation of immunomodulators. A The negative correlation between

TGFB1, CTLA4, IL10, PD1 and their corresponding CpG methylation sites (Spearman correlation). **B** The negative correlation between CD276, VEGFB, PD1 and their corresponding target miRNAs (Spearman correlation). **C** CNV distribution of CD27, CD276, CXCL9, CXCL10, HLA-A, IL2RA and IL10 among three clusters. Each column represents the total proportion of each subtype and each color indicates different type of CNV (Chi-squared test or Fisher's exact test).

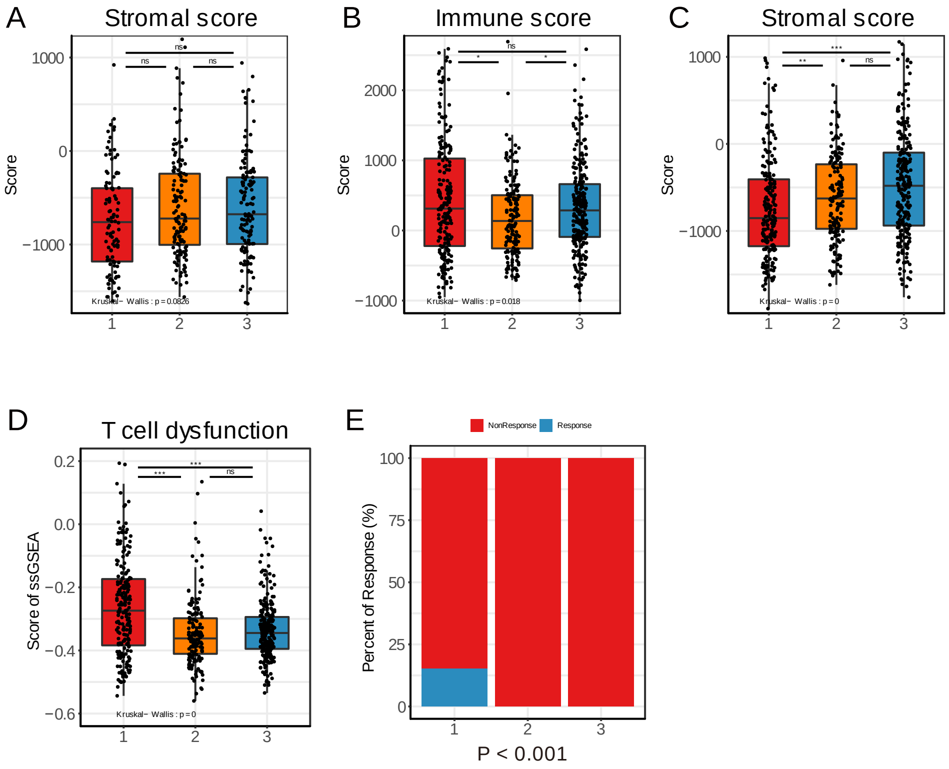

Fig. S12. Evaluation of immune state and prediction of the response to the immune checkpoint blockade therapy. A Stromal score among three subtypes in TCGA-LIHC. **B-C** Immune score and stromal score among three subtypes in meta-validation cohort. **D** T cell dysfunction in meta-validation cohort. **E** Prediction of immunotherapy responsiveness among three clusters in LICA-FR and GSE64041 by TIDE (Fisher's exact test).

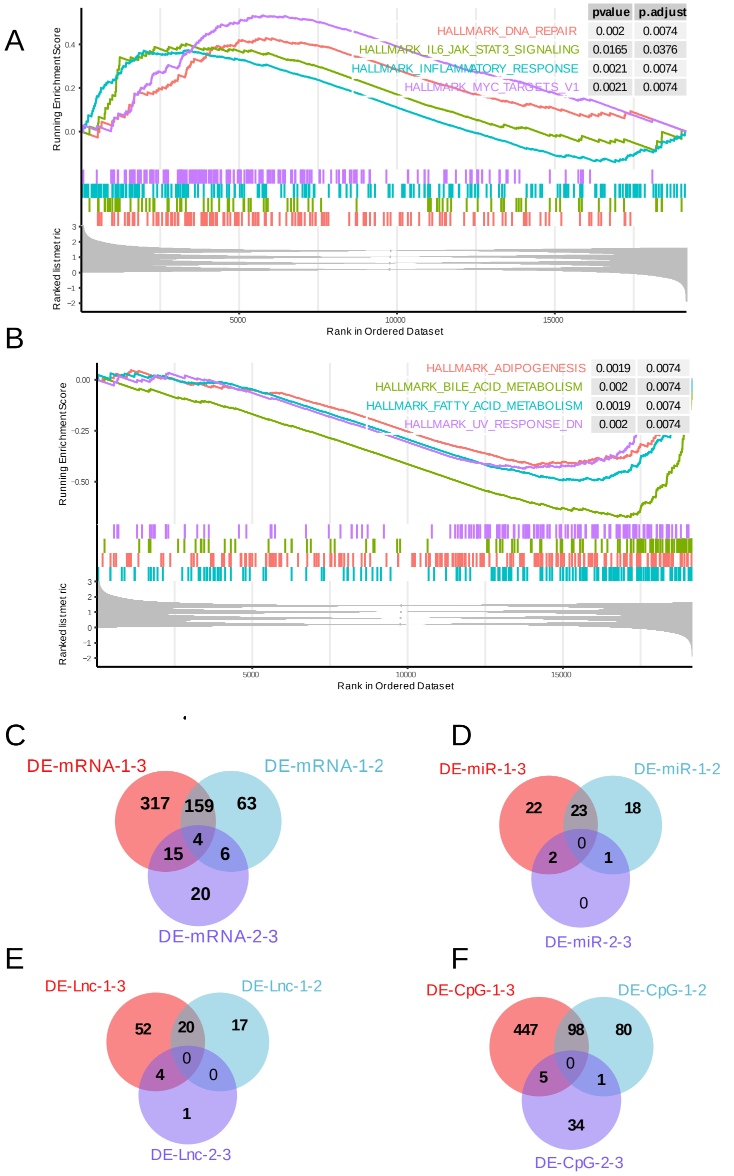

Fig. S13. Gene Set Enrichment Analysis for the cluster1 and cluster3 and multi-omics differential analysis. **A** The enriched Hallmark gene sets in clsuter1. **B** The enriched Hallmark gene sets in clsuter3. **C-F** Schematic diagram of multi-omics differential analysis between any two subtypes. **C** mRNA **D** miRNA **E** LncRNA **F** DNA methylation CpG sites.

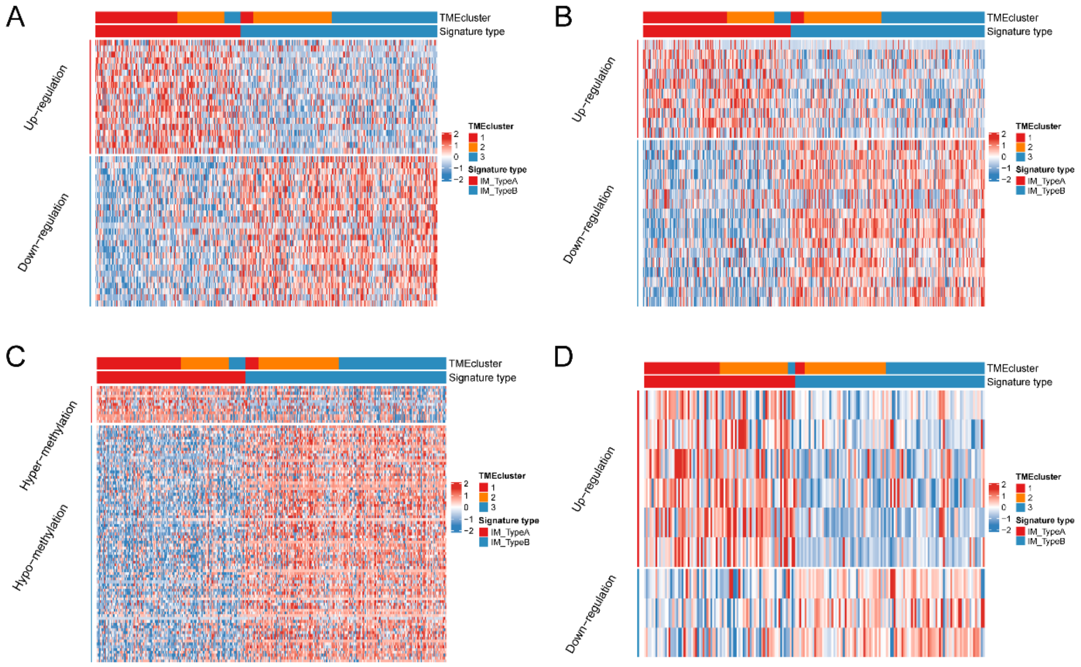

Fig. S14. Heatmap for the featured LncRNA (**A**), miRNA (**B**), DNA methylation CpG sites (**C**), proteins (**D**) (by random forest selecting) distribution in SVM classifier and three clusters.

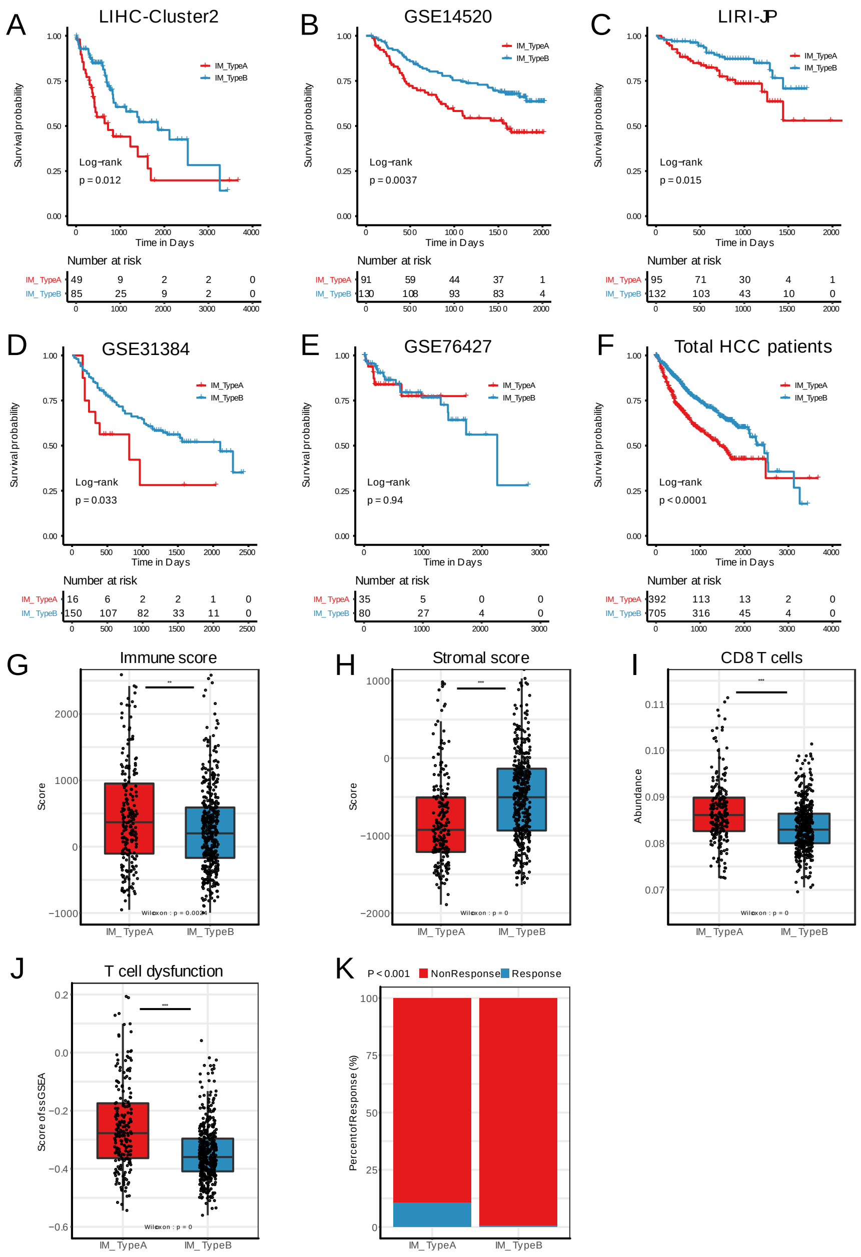

Fig. S15. Prognostic and immunological characteristics of immune subtypes other datasets divided by SVM classifier. Kaplan–Meier OS curves grouped by SVM classifier in TCGA cluster2 (**A**), GSE14520 (**B**), LIRI-JP (**C**), GSE31384 (**D**), GSE76427 (**E**), Whole HCC dataset (**F**). Comparison of immune scores(**G**), stromal scores (**H**), CD8 T cells (**I**), T cell dysfunction (**J**) (Wilcoxon signed rank test) and predicted response to immunotherapy (**K**) (Fisher's exact test) between Type A and Type B based on SVM model in meta-validation cohort.

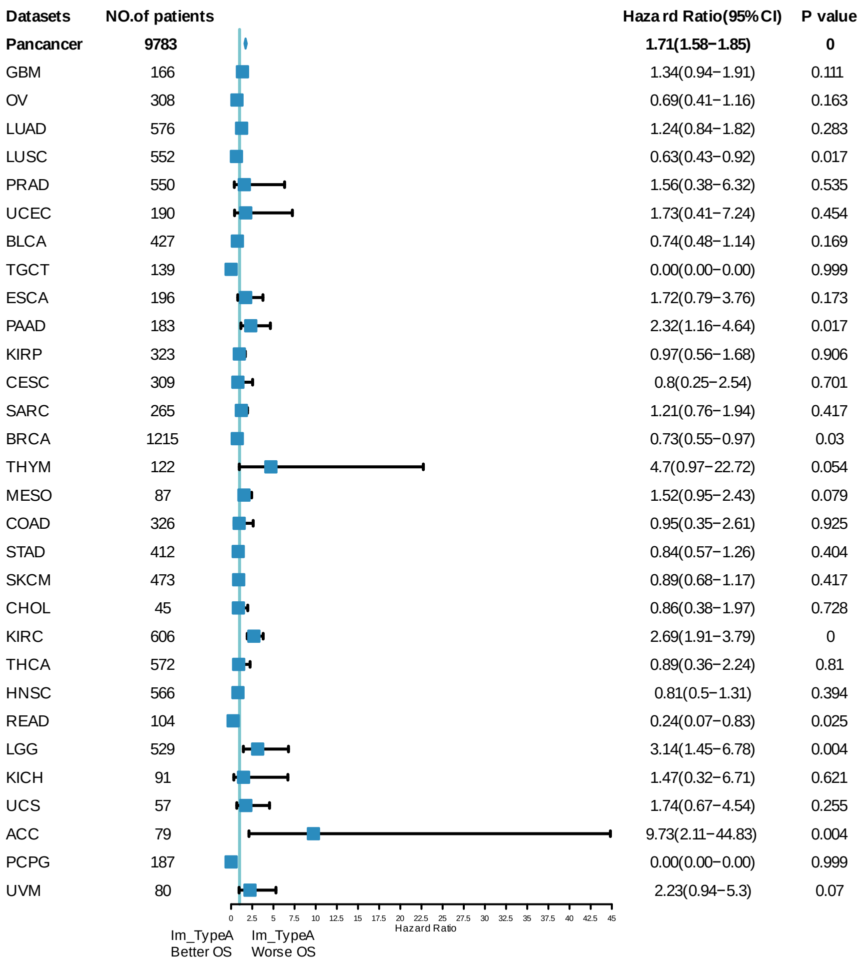

Fig. S16. Forest diagram for subtype analysis between Type A and Type B in Pan-cancer dataset from TCGA.

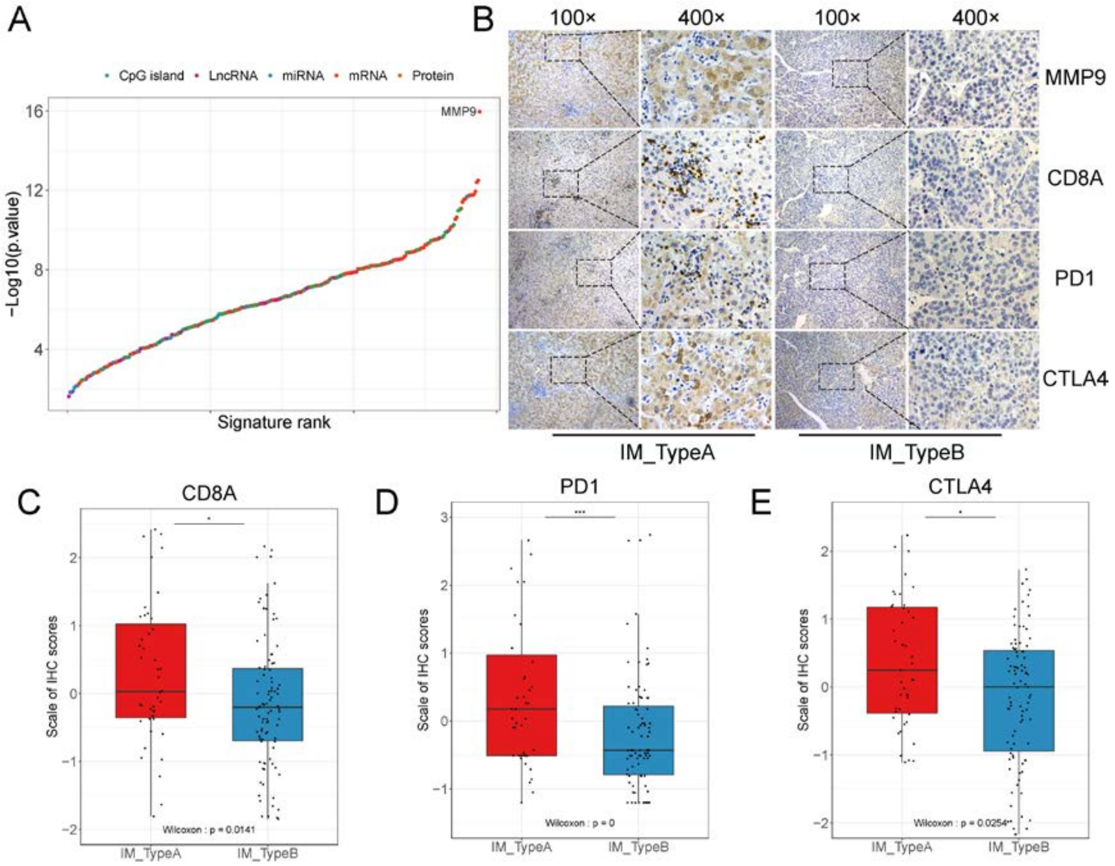

Fig. S17. MMP9 was a potential indicator of HCC immune characteristics. A All multi-omics signatures were arranged from small to large, with MMP9 mRNA having the smallest P value. **B** Representative immunohistochemical images of Type A and Type B showed different expressions of MMP9, CD8A, PD1 and CTLA4(100× and 400×). Comparison of CD8A (**C**), PD1 (**D**), CTLA4 (**E**) (Wilcoxon signed rank test) between Type A and Type B divided by SVM classifier based on normalized immunohistochemical staining scores of MMP9 in Tongji cohort. ns: no significance, *: $P < 0.05$, **: $P < 0.01$, ***: $P < 0.001$.

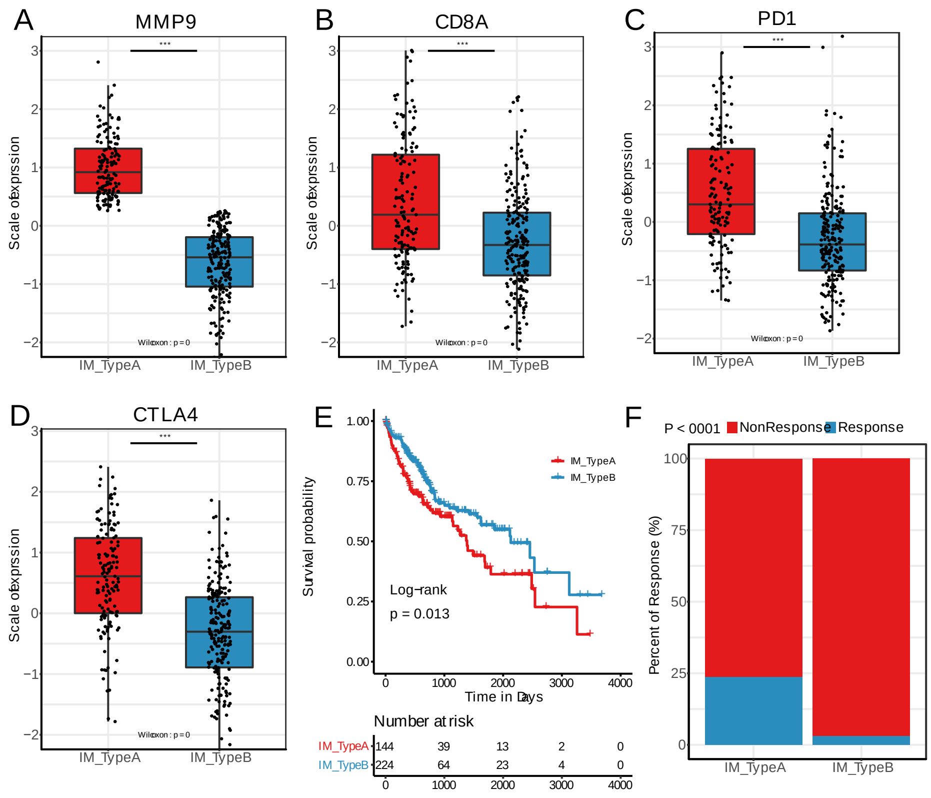

Fig. S18. MMP9 was a potential indicator of HCC immune characteristics. Comparison of MMP9 (**A**), CD8A (**B**), PD1 (**C**), CTLA4 (**D**) (Wilcoxon signed rank test) between Type A and Type B divided by SVM classifier based on gene expression of MMP9 in TCGA-LIHC cohort. **E** Survival analysis of Type A and Type B based on the SVM model in TCGA-LIHC cohort. **F** Predicted response to immunotherapy between Type A and Type B based on MMP9-SVM model. ns: no significance, *: P < 0.05, **: P < 0.01, ***: P < 0.001.
